# Integrated Extraction and Purification of High-Purity Cannabidiol from Industrial Hemp: Continuous Chromatography and Scale-Up Considerations

**DOI:** 10.3390/molecules31213872

**Published:** 2026-10-27

**Authors:** Xin Sui, Dongqing He, Peng Li, Liangyu Zhu, Yang Xie, Tengling Ye, Ling Zhou, Sha Xie

**Affiliations:** 1 Institute of Advanced Technology, Heilongjiang Academy of Sciences, Harbin 150020, China; 2008suixin@163.com (X.S.); lipeng@iathas.ac.cn (P.L.); xyy89650641@126.com (Y.X.); 2 Institute of Natural Resources and Ecology, Heilongjiang Academy of Sciences, Harbin 150040, China; liangyuzhu2025@163.com; 3 MIIT Key Laboratory of Critical Materials Technology for New Energy Conversion and Storage, School of Chemistry and Chemical Engineering, Harbin Institute of Technology, Harbin 150001, China; 4 Key Laboratory of Monitoring for Heavy Metal Pollutants, Ministry of Ecology and Environment, Changsha, 410019, China; darlingsoljah@126.com (L.Z.); shashahjjc0433@163.com (S.X.)

**Keywords:** cannabidiol, industrial hemp, extraction, separation and purification, process integration

## Abstract

Cannabidiol (CBD) is a high-value non-psychoactive constituent of industrial hemp with diverse pharmacological activities. However, its low natural abundance, poor thermal stability, and the difficulty in removing psychoactive Δ^9^-tetrahydrocannabinol (THC) limit the efficient and scalable preparation of high-purity CBD. Although recent reviews cover the cannabis processing chain or chromatographic isolation, a systematic and quantitative comparison of extraction and downstream purification as an integrated workflow is still lacking. This review establishes an integrated comparative framework for producing high-purity CBD from industrial hemp. Conventional solvent extraction, supercritical fluid extraction, physical field-assisted extraction, and green solvent extraction are compared. Mainstream purification approaches, including molecular distillation, chromatography, and crystallization, are discussed, with emphasis on continuous chromatographic strategies such as simulated moving bed (SMB) and recycling chromatography, alongside a critical assessment of their current laboratory/preparative-scale validation and scale-up feasibility. The analysis shows that no single technique can simultaneously balance efficiency, purity, and cost; multi-technology coupling is a promising strategy for future industrial translation. Future research should focus on continuous chromatography, green solvents, process integration, and intelligent manufacturing to bridge the gap between laboratory validation and scalable industrial production.

## 1. Introduction

Industrial hemp (*Cannabis sativa* L.) is an important multipurpose crop [1]. Cannabidiol (CBD) is the major non-psychoactive cannabinoid in industrial hemp, exhibiting prominent pharmacological activities, including antiepileptic, anti-inflammatory, anxiolytic, and anti-tumor effects [2,3,4]. Following the U.S. Food and Drug Administration (FDA) approval of Epidiolex^®^ for the treatment of refractory epilepsy [5], the therapeutic potential of CBD has been formally validated, greatly promoting the global industrialization of hemp-derived pharmaceutical preparations and health-related products [6,7,8]. Currently, many countries have established regulatory frameworks for industrial hemp cultivation and processing [9], driving an increasing demand for green, efficient, and scalable production of high-purity CBD.

Despite growing market demand for CBD, efficient CBD purification still faces multiple technical barriers. First, the natural abundance of CBD in industrial hemp biomass is low, necessitating large-scale processing and increasing production costs. Second, CBD is sensitive to heat and pH changes and is prone to degradation, isomerization, and oxidation under high-temperature or acidic conditions [10], which imposes strict constraints on process parameters. Most importantly, CBD and Δ^9^-tetrahydrocannabinol (Δ^9^-THC, hereafter referred to as THC) are structural isomers with highly similar physicochemical properties, resulting in substantial co-elution during chromatographic separation and poor separation selectivity [11]. This creates a significant challenge for the selective removal of residual THC and leads to an inherent trade-off between product purity and recovery yield [12]. Different jurisdictions enforce distinct statutory limits for THC residues, and these thresholds vary depending on the intended product category and applicable regulatory framework. Therefore, the selective removal of residual THC while preserving high CBD recovery remains the core challenge in CBD refining.

In recent years, several reviews have summarized cannabinoid extraction and purification. Pulido-Bonilla et al. [13] provided a comprehensive overview of the cannabis processing chain, while Pereira et al. [14] focused on chromatographic isolation strategies. In addition, other reviews have addressed extraction methods [15,16], purification and isolation challenges [17,18], correlations between extraction conditions and product efficiency [19], and HPLC-UV analytical approaches [20]. However, a comprehensive review that systematically and quantitatively compares extraction and downstream purification as an integrated workflow is still lacking. To address this gap, this review aims to establish an integrated comparative framework for the production of high-purity CBD. Unlike these reviews, which either cover the broad cannabis processing chain or focus mainly on chromatographic isolation, this review specifically addresses the integrated extraction–purification workflow for high-purity CBD, with particular emphasis on continuous chromatographic strategies and quantitative process comparison.

This review focuses on extraction and purification technologies for the production of high-purity CBD from industrial hemp. The distribution and physicochemical properties of CBD, as well as the major impurities present in industrial hemp, are first outlined. Conventional solvent extraction, supercritical fluid extraction, physical field-assisted extraction, and green-solvent extraction are then systematically compared in terms of working principles, applicability, and current scale-up limitations. Building on this comparison, the stage-wise functions of molecular distillation, crystallization, and chromatography within the purification cascade are discussed, with particular emphasis on chromatography-centered multi-technique coupling and continuous chromatographic strategies such as simulated moving bed (SMB) and recycling chromatography. A quantitative comparison framework for purification techniques is further established, integrated purification routes are proposed, and their applicability is evaluated, particularly in terms of their laboratory/preparative-scale validation status. Finally, key bottlenecks and future research directions are summarized, including continuous chromatography, green-solvent recovery, and intelligent manufacturing. With this structure, this review aims to provide an integrated comparative framework and critical insights to bridge the gap between current laboratory-scale validation and the future industrial translation of high-purity CBD production.

## 2. Physicochemical Properties of CBD

The structural differences between CBD and THC, the physicochemical properties of CBD, and its chemical speciation in industrial hemp constitute the essential theoretical foundation for the design of extraction and purification processes. Elucidating these fundamental issues is critical for developing subsequent extraction and separation technologies.

### 2.1. Structure and Properties of CBD

**Figure 1:**
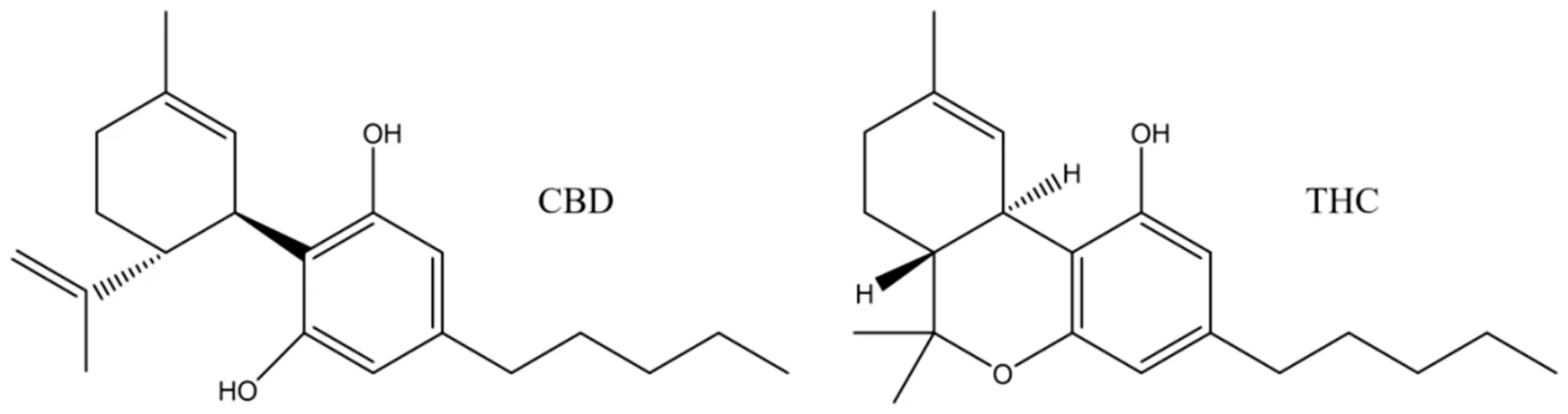
Structural comparison of isomeric CBD and THC.

CBD has a molecular formula of C_21_H_30_O_2_ and a relative molecular mass of 314.46. Its structure contains two phenolic hydroxyl groups and a cyclohexene ring, which classifies it as a monoterpenophenol compound [21]. CBD and THC are isomers, sharing considerable similarities while presenting notable disparities in molecular skeleton, functional groups, and stereochemistry (Figure 1). CBD bears an open ring framework, in which the cyclohexene moiety is tethered to the phenolic ring via an alkyl side chain. In contrast, THC possesses a tricyclic molecular skeleton fused through an intramolecular oxygen bridge. These structural similarities result in remarkably analogous physicochemical properties (e.g., polarity and solubility) and nearly identical chromatographic retention behavior. Such overlapping characteristics severely impede their effective separation, forming a core technical bottleneck for the large-scale production of high-purity CBD.

In terms of physicochemical properties, CBD is a white to off-white crystalline powder at room temperature. It exhibits pronounced lipophilicity and is readily soluble in organic media including ethanol, methanol, n-hexane and chloroform, yet remains sparingly soluble in aqueous matrices [22]. Meanwhile, CBD possesses poor chemical stability and tends to oxidize or degrade under heat, illumination, acidic and alkaline surroundings. Notably, thermal isomerization of CBD to THC is a critical side reaction that must be strictly controlled during purification processes [10,23].

Overall, the strong lipophilicity of CBD dictates the use of organic solvent extraction, while its thermal sensitivity restricts the processing temperature window. Furthermore, the structural similarity to THC imposes stringent requirements on the selectivity of separation media.

### 2.2. Chemical Speciation of CBD

In industrial hemp, CBD exists predominantly as its acidic precursor, CBDA, along with minor amounts of neutral CBD [24]. CBD is distributed unevenly across different parts of the plant, with the highest concentrations found in female inflorescences, pistils, and top leaves, whereas stalks and seeds contain relatively low levels, and no cannabinoids are detectable in the roots [25]. CBDA possesses low binding affinity for cannabinoid receptors (CB1/CB2) and must undergo thermally induced decarboxylation to be converted into CBD to exert pharmacological effects [26]. CBDA features relatively high polarity together with inferior stability. It is prone to decarboxylation, yielding CBD during drying, thermal treatment and extraction. Furthermore, cannabis cultivars, cultivation environments, harvest timing and processing approaches exert influences on the CBDA/CBD ratio and total cannabinoid concentration in plant tissues [27,28].

Understanding the natural occurrence, tissue distribution, and decarboxylation rules of CBD is essential for guiding raw material screening, pretreatment design, and the construction of an integrated extraction–purification process.

## 3. Extraction Technologies

Efficient extraction of CBD from industrial hemp constitutes a critical step governing the feasibility of subsequent purification and the overall process economics. The selection and optimization of extraction technologies demand comprehensive consideration of various factors, including feedstock properties, extraction yield, solvent safety, and the complexity of downstream purification. In the following sections, recent advances in CBD extraction technologies are systematically reviewed from two aspects: raw material pretreatment and extraction approaches.

### 3.1. Raw Material Pretreatment

Raw material pretreatment aims to modify the physical morphology and chemical composition of the plant matrix via physical or chemical strategies, thus establishing favorable conditions for subsequent extraction. Main operations include harvesting timing control, drying, comminution and decarboxylation.

#### 3.1.1. Harvesting, Cleaning, Drying and Grinding

Harvest timing directly influences the accumulation of secondary metabolites in industrial hemp. CBDA is the dominant cannabinoid, and its biosynthesis exhibits obvious temporal and spatial regularity. Its concentration in female inflorescences and bracts changes dynamically along with plant growth stages. Related experiments verify that the peak period of cannabinoid accumulation ranges from full bloom to the initial grain-filling stage [29]. Premature harvesting yields low biomass and insufficient cannabinoid biosynthesis. In contrast, delayed harvesting is affected by temperature decline and plant senescence; the CBDA concentration rises first and then falls. Meanwhile, acidic cannabinoids readily undergo spontaneous decarboxylation, increasing THC levels [30].

Impurity removal and drying should be implemented promptly after harvesting [31]. The elimination of impurities such as stems, aged leaves and dust can prevent unnecessary consumption of extraction solvents and avert color and flavor defects in final products [32]. Drying methods exert a direct influence on cannabinoid retention rates. Open-air natural sunning is susceptible to weather fluctuations and may trigger material mildew. Various controllable artificial drying technologies have been developed to overcome such drawbacks. Comparative studies on multiple drying strategies, including freeze drying, infrared-hot air combined drying, and gradient non-isothermal hot air drying have verified that hot-air drying at 40–60 °C or shade drying can effectively inhibit the photooxidation and thermal degradation of CBDA. This approach maximizes the retention of active constituents while enabling bacteriostasis and modulation of enzyme activity. Temperatures above 60 °C accelerate excessive decarboxylation of CBDA, resulting in the loss of target cannabinoids. Although short-time high-temperature infrared treatment accelerates drying speed, it leads to substantial CBD loss. Gradient-temperature drying can slightly elevate total CBD content yet brings markedly increased process complexity [33].

Particle size after grinding is an important parameter that influences extraction mass transfer efficiency. Grinding breaks plant cellular structures and increases the specific surface area, thereby promoting solvent penetration and the release of target components. However, overly fine particles lead to extensive co-extraction of impurities such as chlorophyll and waxes, complicating subsequent purification. Moreover, material channeling readily occurs, lowering extraction efficiency. Therefore, an appropriate particle size strikes a balance between extraction rate and selectivity to achieve efficient recovery of the target components [34].

#### 3.1.2. Decarboxylation

**Figure 2:**
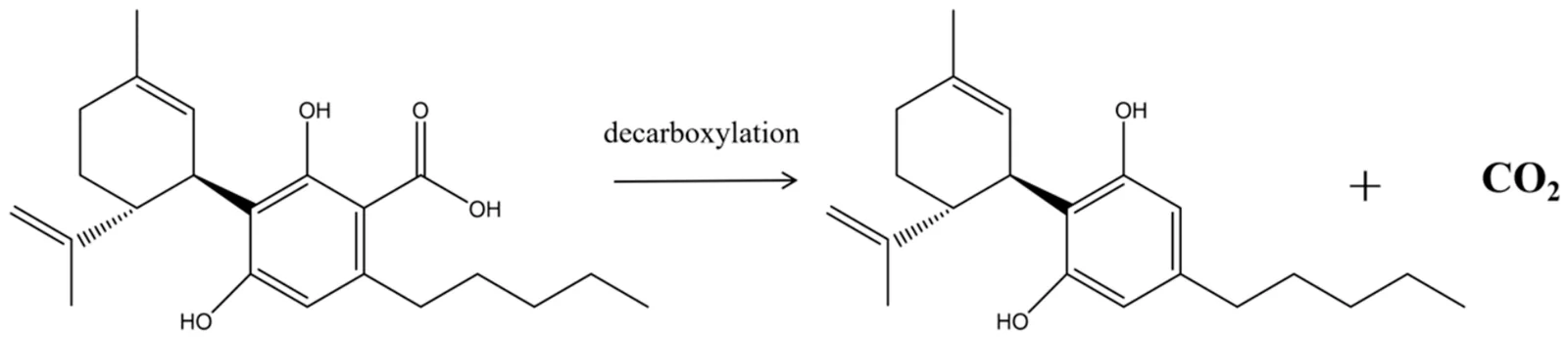
Decarboxylation reaction of CBDA to CBD.

The decarboxylation of CBDA to CBD proceeds via the thermal or photochemical elimination of the side-chain carboxyl group as CO_2_ (Figure 2). Because this reaction is governed by both temperature and duration, higher temperatures accelerate the reaction but also increase the risk of side reactions, necessitating a trade-off between conversion efficiency and thermal degradation [35]. It has been established that decarboxylation proceeds efficiently at 120 °C for 60 min or 140 °C for 30 min [36]; a rapid alternative at 160 °C for 10 min is also effective for dried floral-leaf materials [37]. However, these optimized protocols are not without upper limits. It should be noted that excessively high temperatures or prolonged heating tend to cause oxidative degradation of CBD, and sustained heating at 175 °C has been shown to further promote intramolecular cyclization to THC [10].

Based on industrial-manufacturing considerations, decarboxylation can be categorized into three routes: pre-extraction, in situ simultaneous-extraction, and post-extraction decarboxylation.

Pre-extraction decarboxylation thermally treats dried inflorescences using oven or microwave heating. Oven decarboxylation suffers from high-energy consumption, uneven heat distribution and long processing cycles. Microwave technology achieves uniform heat transfer via dielectric heating and shortens treatment duration remarkably, delivering better pretreatment efficiency. Design-Expert-assisted optimization of oven-based decarboxylation was carried out for different hemp cultivars. When reaction time served as the key variable, the optimal conditions were 149 °C for 41 min for high-CBD feedstock and 137 °C for 57 min for high-THC feedstock. With relaxed time constraints, a unified isothermal condition of 131 °C can be applied, with holding times of 102 min and 65 min respectively. Kinetic data reveal that loading capacity at elevated temperatures strongly influences decarboxylation rates, and THCA conversion varies among cultivars. These results provide process references for large-scale oven production complying with GMP requirements [38]. Dedicated microwave devices with rotating chambers handle 150 g of inflorescence per batch. Homogeneous decarboxylation is achieved at 120 °C within 30 min. The closed chamber also reduces terpene loss and suppresses pungent-odor emission [39].

Simultaneous-extraction and decarboxylation complete CBDA conversion by utilizing the inherent thermal conditions of extraction systems. For supercritical CO_2_ extraction, coupling of decarboxylation and extraction is realized when the system temperature rises above 80 °C. A vacuum-microwave olive-oil trapping system maintains 120 °C under mild vacuum for 30 min. It completes acidic-cannabinoid decarboxylation and volatile-terpene recovery in one operation, integrating multiple unit steps and simplifying production workflows [40].

Post-extraction decarboxylation performs thermal conversion on crude CBD oleoresin. Decarboxylation can also be coupled with downstream separation units. A preheating module installed upstream of molecular distillation completes decarboxylation before materials enter the high-vacuum chamber, enabling integrated decarboxylation-molecular-distillation operation. This pre-separation decarboxylation reduces residual CBDA and improves CBD purity [41].

In summary, decarboxylation is the essential unit for converting CBDA into CBD. Pre-extraction, simultaneous-extraction, and post-extraction decarboxylation can be flexibly selected according to production capacity and supporting facilities, each suitable for distinct industrial scenarios. The adoption of emerging approaches such as microwave-assisted decarboxylation and molecular-distillation-coupled decarboxylation offers diverse strategies for developing efficient, mild and controllable decarboxylation systems.

### 3.2. Extraction Methods

**Figure 3:**
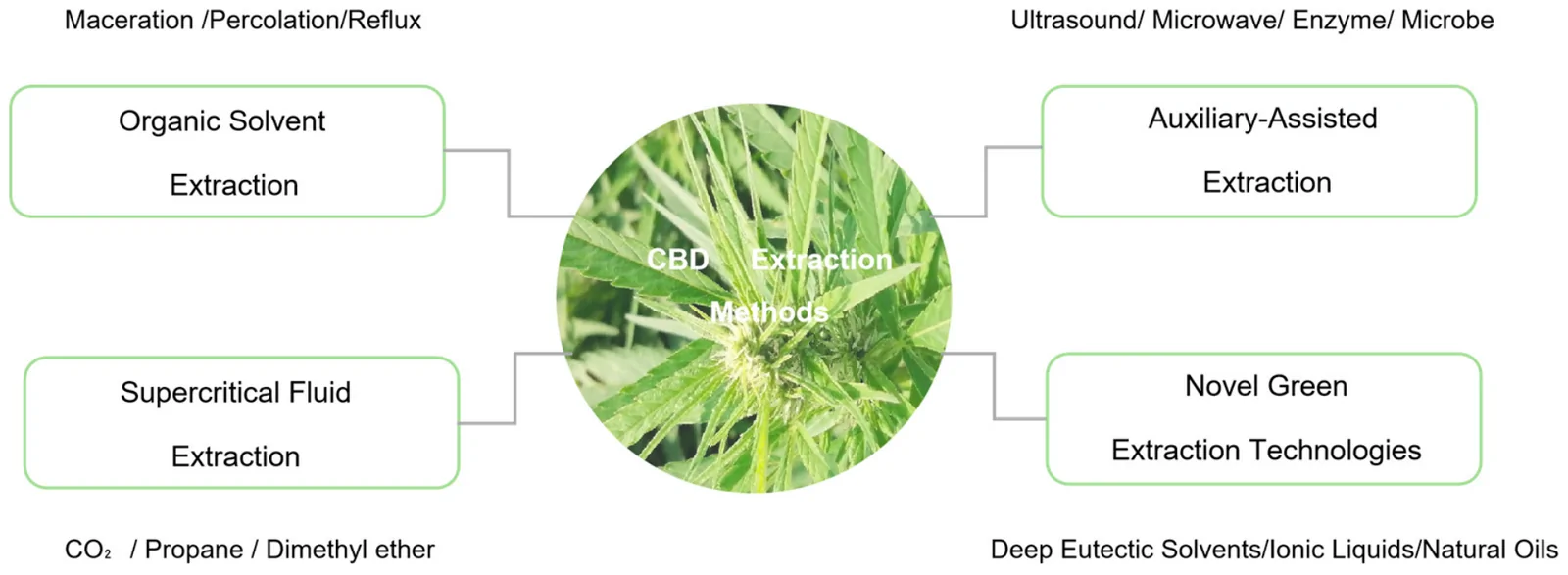
Classification of CBD extraction methods.

Following raw material pretreatment and decarboxylation, the choice of extraction methods determines cannabinoid recovery, crude product purity, and overall product quality. Four major approaches are commonly employed for CBD production: organic solvent extraction, supercritical fluid extraction, field-assisted extraction, and emerging green-solvent extraction (Figure 3).

#### 3.2.1. Organic Solvent Extraction

Organic solvent extraction is the most well-established technique for crude CBD extraction. Solvent selection is a fundamental aspect of organic-solvent extraction [42], whereas process compatibility and product stability are key considerations for scale-up [43]. Alcohols, alkanes, and ethers are widely employed for cannabinoid extraction. These methods feature operational simplicity, good equipment compatibility, and straightforward scale-up potential. Nevertheless, solvent-dependent dissolution selectivity varies greatly, which directly determines the impurity load of crude extracts and, consequently, the downstream purification burden and associated costs.

Ethanol is a widely used green solvent for cannabinoid extraction [43] because it enables simultaneous extraction of multiple cannabinoids (e.g., CBD and CBDA) and has a favorable safety profile [44]. However, it also co-extracts polar impurities such as chlorophyll and polysaccharides, which often necessitate low-temperature winterization and increase downstream costs. Despite this drawback, optimized cold ethanol extraction has been demonstrated at the pilot scale using 3 kg biomass, achieving an extract yield of 14.62%, with 50.58% CBD, 24.89% CBDA, and 2.41% THC under conditions of −31 °C, 33 min extraction time, and 1.8% (*w*/*v*) solid loading [45]. In contrast, nonpolar solvents (propane, butane, n-hexane, diethyl ether) offer higher selectivity and lower pigment content but are limited by solvent residues and flammability hazards [43]. Pentane and diethyl ether exhibit better selectivity than ethanol; pentane afforded the highest cannabinoid content in the combined extract (52.9% at 21 °C for 15 min) [46]. This approach, however, remains confined to laboratory scale, where solvent recovery and operational safety become major constraints upon scale-up.

Among conventional approaches (maceration and heating reflux), heating reflux generally provides higher extraction yields than maceration for the recovery of CBD and CBG from hemp [47]. Although heating reflux is typically performed below 100 °C, CBD may undergo isomerization and thermal degradation under harsher high-temperature conditions, such as those encountered in pressurized solvent extraction [48]. Solvent polarity markedly affects CBD stability: low-polarity systems such as n-hexane suppress degradation side-reactions, and appropriate water content can further enhance CBD stability [48]. Accordingly, practical solvent selection should strike a balance between extraction recovery and product stability.

In general, process parameters (temperature, extraction duration, solid–liquid ratio, and extraction cycles) exert significant influences on extraction efficiency. Organic-solvent extraction faces three major challenges: substantial co-extraction of impurities, high thermal lability of active components, and inherent risks related to organic solvent residues [43]. Current research focuses on multi-parameter collaborative optimization and targeted development of low-impurity extraction systems. These efforts aim to improve extraction performance and satisfy stringent requirements for the scaled-up production of high-purity CBD.

#### 3.2.2. Supercritical Fluid Extraction

Supercritical CO_2_ (scCO_2_) extraction takes advantage of gas-like diffusivity and liquid-like solvating power above the critical point. Tuning temperature and pressure modifies fluid solvation performance to achieve selective solubilization of cannabinoids [49]. Studies demonstrate that scCO_2_ extracts cannabinoids from both raw and decarboxylated industrial hemp materials. Crude extract mass yields range from 5.8% to 12.0%, with total cannabinoid recovery of 51–100%. Under an extraction pressure of 200 bar, the maximum CBD mass fraction in crude extracts reaches 44.9% [50]. The process leaves no solvent residue, and its low-temperature operation reduces degradation risks for thermolabile components. The tunable extraction selectivity also allows combination with winterization-based impurity removal, making scCO_2_ a viable technical route for high-quality crude CBD preparation [51].

Raw material pretreatment and process parameter tuning are critical for desirable scCO_2_ extraction performance. Although decarboxylation slightly reduces overall crude extract yield, it improves neutral cannabinoid recovery, because neutral cannabinoids exhibit higher solubility in scCO_2_ than their acidic precursors. Pure scCO_2_ preferentially dissolves lipophilic neutral cannabinoids yet shows limited capacity for more polar acidic cannabinoids. Adding 5% ethanol as a co-solvent or raising extraction pressure improves fluid polarity for simultaneous extraction of neutral and acidic cannabinoids. A two-step separation strategy can preliminarily fractionate extracts and alleviate downstream purification loads.

Nevertheless, scCO_2_ extraction has notable practical drawbacks. High capital investment and maintenance costs restrict batch-processing capacity, limiting large-scale continuous production. Moreover, CBD and THC exhibit highly similar solubility behaviors in scCO_2_. Isolation of the two compounds cannot be accomplished by extraction alone; high-resolution downstream purification, such as preparative chromatography, is required to obtain high-purity products [52]. This process is highly sensitive to operating conditions; minor fluctuations of 1 °C or 1 bar can significantly alter CO_2_ density and diffusion coefficient, thereby shifting extraction selectivity and recovery.

Besides CO_2_, high-pressure solvents including propane and dimethyl ether have also been explored for cannabinoid extraction. These solvents differ distinctly from scCO_2_ in solubilizing capacity and component selectivity. They serve as complementary extraction systems and expand the technical options for different feedstock properties and product specifications [50].

#### 3.2.3. Field-Assisted Extraction

Conventional approaches such as maceration and thermal reflux suffer from substantial mass-transfer resistance and long processing durations. Elevated operating temperatures further trigger thermal degradation of heat-sensitive cannabinoids, including CBD and CBDA. Field-assisted extraction employs physical fields (ultrasound, microwave) to intensify mass transfer and cell disruption, enabling improved extraction efficiency and reduced solvent consumption under mild conditions. For industrial-hemp-derived CBD extraction, ultrasound-assisted extraction (UAE) and microwave-assisted extraction (MAE) represent the most widely investigated techniques, which differ markedly in their underlying mechanisms, process constraints and, most critically, their potential for industrial scale-up.

(1) Ultrasound-assisted extraction

Ultrasonic cavitation and micro-turbulence enhance solid–liquid mass transfer, enabling efficient solubilization of CBD and its acidic precursor CBDA under low-temperature and short-duration conditions [53,54]. Compared with thermal reflux, UAE is better suited for extracting heat-labile cannabinoids. Most existing UAE investigations for industrial-hemp-derived CBD are carried out at the lab-scale for process screening and modeling. Response surface methodology (RSM) and artificial neural networks (ANN) are widely adopted to interpret the interactive effects of ultrasonic power, temperature, extraction time, solid–liquid ratio and solvent composition. Ethanol-water mixtures are the most widely adopted extraction medium in this field, favored for their lower environmental impact and ease of solvent recovery [55].

Solvent composition strongly governs extraction selectivity and recovery. A 50% ethanol-water system has proven effective for the simultaneous solubilization of cannabinoids and polyphenols. For the Helena hemp cultivar, UAE gave a higher extraction yield (17.54% vs. 15.28%) and CBD content (0.8752 vs. 0.4310 mg/mL) than conventional maceration under identical conditions, while reducing extraction time from 24 h to 1 h [56]. Harvest timing must also match process parameters: aerial tissues collected at early-flowering stages favor polyphenol accumulation, whereas full-flowering biomass contains higher intrinsic cannabinoid levels. This work further showed that ANN delivers superior multi-objective prediction performance compared with RSM, though both models are built exclusively on laboratory-scale datasets. Jaroenkunpanit et al. [53] revealed component-specific mass-transfer behavior during UAE via kinetic-thermodynamic analysis. CBD follows first-order kinetics, THC obeys the power-law model, while polyphenols fit the two-stage Peleg model. Broad confidence intervals for several thermodynamic parameters indicate gaps in current mechanistic understanding.

UAE shortens extraction cycles from tens of hours to tens of minutes and features low capital requirements for small-batch preparation workflows. Nevertheless, the scale-up of UAE remains a critical engineering bottleneck. Optimal lab-derived parameters cannot be directly transferred to large-volume systems. Increased reactor volume produces uneven acoustic-field distribution and energy attenuation, which bring about batch-to-batch variability. Reactor concepts such as multi-frequency probe arrays and circulating fluidized beds have proven capable of mitigating such drawbacks in general chemical engineering practice [57]. However, empirical validations targeting industrial-hemp CBD extraction remain scarce [54]. Moreover, most RSM-based optimizations pursue only maximum CBD yield. As highlighted by Jaroenkunpanit et al., practical deployment requires multi-objective optimization that balances CBD output, THC control and polyphenol retention.

A frequently overlooked concern is the cascading effect of co-extracted impurities. While ultrasonic cell disruption releases target cannabinoids, it also promotes dissolution of waxes, chlorophyll, and other non-target polyphenolic components. Current studies mostly limit characterization to total phenol and total flavonoid quantification. Few works quantitatively assess how elevated impurity loads impact downstream winterization and column-chromatography operations. Quantitative economic evaluations linking higher impurity levels to shortened resin service life and increased eluent consumption are particularly lacking. This restricts techno-economic assessment covering the whole extraction-purification workflow. Additionally, local hotspots generated by acoustic cavitation may trigger uncontrolled decarboxylation of CBDA [58]. Large energy-output discrepancies across different equipment create widely scattered optimal power-temperature windows in published literature [59]. Standardized process parameters transferable across apparatus are yet to be established. Existing kinetic and thermodynamic models can describe mass-transfer mechanisms, yet their underlying databases originate solely from lab-scale tests. Parameter calibration for scaled-up operating conditions remains insufficient, representing a critical hurdle to the industrial implementation of UAE.

(2) Microwave-assisted extraction

Microwaves generate volumetric heating in plant materials via dipole rotation and ionic conduction, rapidly disrupting glandular trichomes and cell walls to enhance solid–liquid mass transfer, thereby shortening extraction time and reducing solvent consumption [60]. Most MAE studies on industrial hemp are conducted at laboratory batch scale, typically employing RSM for parameter optimization [61,62]. While MAE offers marked advantages in extraction speed, its transition from lab to industrial scale faces distinctive challenges.

Solvent selection largely determines the profile of extracted components. Pure-water MAE effectively recovers polar polyphenols but yields limited CBD due to the poor solubility of lipophilic cannabinoids in water; raising temperature improves CBD solubilization at the cost of potential flavonoid degradation. A bio-based candidate solvent, 2-methyltetrahydrofuran (2-MeOx), enables concurrent recovery of CBD, polyphenols and terpenes. Its lower latent heat of vaporization compared with ethanol reduces energy consumption for solvent recycling. For example, water-saturated 2-MeOx (4.5% water) increased CBD recovery to 81.30 mg/g DM compared with 75.45 mg/g DM for dry 2-MeOx and 77.71 mg/g DM for ethanol. Optimized MAE with 2-MeOx (1000 W, 20 min, 1.72% water) achieved 86.76 mg CBD/g DM, which was about 15% higher than conventional extraction [63]. However, one-step microwave-driven decarboxylation is less efficient in 2-MeOx than in ethanol systems [63]. Ethanol-water mixtures remain the most widely adopted system, offering a balanced trade-off among CBD yield, decarboxylation efficiency and process compatibility. These three solvent systems each present distinct trade-offs, and current literature does not permit a definitive judgment as to which achieves the optimal balance among recovery yield, product integrity and energy consumption.

Two major challenges constrain MAE scale-up. First, microwave hot-spot effects induce uncontrolled CBDA decarboxylation and thermal degradation of heat-sensitive CBD, terpenes and polyphenols. Discrepancies in electromagnetic field distribution between single-mode and multi-mode reactors further cause severe scale-up distortion. Second, quantitative assessments of the impurity-related burden on winterization and column chromatography under large-scale conditions are scarce, hindering the establishment of an economic link between extraction performance and downstream purification costs. Pilot-scale continuous-flow MAE systems using 915 MHz microwave sources have been reported for industrial-hemp processing, yet fully documented industrial-scale cases remain rare in the peer-reviewed literature [64]. Single-mode continuous-flow microwave reactors remain at the proof-of-concept stage, constrained by material clogging and non-uniform solid–liquid mixing.

A comparison of UAE and MAE shows that both shorten extraction time relative to conventional methods. UAE is milder, typically using ethanol–water, and is suitable for heat-sensitive cannabinoids, whereas MAE is faster due to volumetric heating and efficient cell disruption. However, most studies for both techniques remain at laboratory scale, and systematic scale-up and continuous-flow studies are scarce. Therefore, UAE and MAE are complementary rather than interchangeable, and the choice depends on the target product, feedstock, and production scale. Future efforts should focus on pilot-scale validation and integrated process design.

#### 3.2.4. Emerging Green-Solvent Extraction

In recent years, CBD extraction technologies for industrial hemp have trended toward greener, more efficient, lower-toxicity, and environmentally friendly processes. Various emerging green extraction techniques have been developed and continuously optimized. These approaches mitigate many drawbacks associated with conventional extraction methods. This section summarizes recent research progress, key merits and existing challenges of green extraction technologies, including deep eutectic solvents, ionic liquids and natural oils.

Deep eutectic solvents (DES) are formed by combining hydrogen-bond acceptors (HBA) and hydrogen-bond donors (HBD). DES are often considered green solvents owing to their low volatility and generally low toxicity. However, these attributes depend strongly on the selected HBA and HBD combinations and cannot be generalized to all DES systems. For example, among 24 choline chloride-based DESs tested, only two were classified as readily biodegradable [65]; replacing glucose with oxalic acid as the HBD shifted the DES from low-hazard to moderate-hazard classification (EC_50_: >10 mM vs. 1.64 mM) [66].

Terpenes, saccharides and natural organic acids have been applied to synthesize both hydrophilic and hydrophobic DES for recovering bioactive compounds from industrial hemp. Solvent type and extraction time are identified as key factors governing extraction efficiency. Solvent-screening experiments indicate that menthol-lauric acid DES at a molar ratio of 2:1 delivers the best extraction performance for CBD and CBDA. Its total extraction yield reaches (11.07 ± 0.37) mg/g, which is markedly higher than other DES and ethanol. This system achieves higher extraction yields and suppresses the co-extraction of impurities such as chlorophyll and waxes, thus providing improved extraction selectivity [67]. However, this result is mainly based on specific feedstocks and laboratory-scale tests, and its applicability to different industrial hemp varieties, scaled-up processes and continuous extraction still requires further verification.

Despite these advantages, most DES exhibit high viscosity originating from extensive hydrogen-bond networks. This inherent property reduces CBD diffusivity and slows extraction kinetics; a viscosity threshold of approximately 500 mPa·s has been reported for biomass extraction systems, beyond which mass transfer is severely limited [68]. Solvent recovery is also challenging because the extremely low volatility of DES prevents conventional distillation, and extraction performance declines after repeated cycles [69]. Moreover, the strong solvation interaction between DES and cannabinoids complicates downstream separation to obtain high-purity CBD. For example, after seven cycles, a choline chloride: p-toluenesulfonic acid (ChCl: pTSA) DES exhibited a reduction in dissolution efficiency from nearly 100% to below 70% due to proton depletion and water accumulation [69].

Owing to their overall low volatility and potential cost advantages, DES represent promising alternatives to conventional organic extraction solvents. However, their practical application in CBD extraction still requires case-specific evaluation of viscosity, solvent recovery, recyclability, downstream separation and toxicological risks [70].

Ionic liquids (ILs) are low-temperature molten salts composed of organic cations and inorganic/organic anions that remain liquid below 100 °C. They exhibit tunable polarity, strong hydrogen-bond interactions and extremely low volatility. ILs can disrupt plant cell walls to promote the release of intracellular bioactive components, with excellent selectivity and stability for target extracts. ILs simultaneously achieve extraction, biochemical transformation and antioxidant effects. They facilitate the isomerization of CBDA to CBD and inhibit CBD degradation. Under optimized conditions, the dry-weight CBD extraction yield reaches 7.23 mg/g, which is significantly higher than the 4.56 mg/g obtained via conventional methanol extraction [71].

Despite these advantages, IL-based extraction still faces several practical limitations. Many ILs exhibit high viscosity, which reduces mass-transfer efficiency and complicates pumping, mixing and phase separation at larger scales [72]. Their extremely low volatility prevents distillation-based recovery and complicates downstream separation of CBD from the IL phase [73]. Recycling of ILs is often limited; extraction efficiency declines after repeated cycles, and accumulated impurities can alter solvent performance [73]. Residual ILs in the final product may also raise safety and regulatory concerns, particularly for pharmaceutical applications [74]. In addition, conventional imidazolium- and pyridinium-based ILs with halide-containing anions are known to be highly toxic and poorly biodegradable; therefore, the “green” label should be used with caution [74]. Moreover, single IL extraction often requires secondary separation with organic solvents, which further weakens the overall environmental advantage.

To optimize extraction performance and integrate the advantages of different green technologies, researchers have constructed a synergistic ionic liquid-supercritical CO_2_ (IL-SFE) extraction system. Using 1-ethyl-3-methylimidazolium acetate and choline acetate as media, feedstocks are pretreated at 70 °C for 15 min. After dilution of ILs with water at a ratio of 1:3, supercritical CO_2_ extraction is performed at 20 MPa and 70 °C for 2 h. This protocol achieves a maximum CBD extraction yield of 15.6 mg/g, superior to that of traditional ethanol extraction [75].

The synergistic IL-SFE system presents outstanding extraction efficiency and can replace conventional solvent extraction and co-solvent-assisted supercritical extraction. However, the reported ILs have so far been recycled only under laboratory conditions, and the number of cycles, recovery efficiency and purity of the recycled ILs require further quantification [73]. The environmental burden of IL synthesis and purification should also be included in the overall assessment. Therefore, although this strategy provides a promising route for the green preparation of plant secondary metabolites, its industrial-scale application still requires techno-economic and life-cycle validation [76].

Extraction of CBD using natural plant-derived oils as carriers represents an important research direction for the green recovery of bioactive components from industrial hemp. Most available studies adopt olive oil and medium-chain triglycerides as extraction media. Physical intensification strategies such as ultrasound and microwave assistance are applied to greatly improve mass-transfer efficiency and extraction rates of cannabinoids. Related investigations demonstrate that optimized probe-ultrasound treatment (2 mm or 7 mm probe) achieves comparable extraction performance within 10 min, which matches the result of 40 min thermal maceration [77]. Characterizations, including detection of volatile carbonyl compounds, oxidized/conjugated fatty acids, tocopherols and their oxidation products, together with differential scanning calorimetry (DSC) analysis [78], reveal that ultrasound exerts no obvious adverse impacts on oil quality. The overall properties of treated oils remain largely consistent with those of the starting feedstock oils [77]. Owing to the strong lipophilicity of cannabinoids, efficient separation between target cannabinoids and oil carriers is difficult to accomplish in oil-based extraction systems. Cannabinoids mostly remain as oil-soluble complexes in final products. These oil-loaded extracts can be directly utilized without further purification. They are compatible with multiple application scenarios, such as oral formulations, functional foods and skin-care cosmetics. Featuring green operation, low energy consumption and simple workflow, this technique shows prominent advantages for industrial translation.

#### 3.2.5. Comprehensive Comparison and Commentary on Different CBD Extraction Technologies

This section systematically reviews four categories of extraction technologies: conventional organic-solvent extraction, supercritical-fluid extraction, field-assisted extraction, and emerging green-solvent extraction. Table 1 summarizes the representative processes, extraction time, yield/recovery, solvent consumption, reported experimental scale, along with the key advantages and limitations of the four extraction categories. Most studies were conducted at laboratory scale; therefore, the data should be regarded as indicative ranges rather than universally applicable specifications.

Among the four extraction categories, conventional ethanol maceration and supercritical CO_2_ extraction are currently the most widely used in industrial practice. Ethanol maceration is simple and well-established but requires long extraction times and co-extracts substantial impurities, increasing the downstream purification load [43,46]. Supercritical CO_2_ extraction offers low impurity levels and solvent-free operation but requires high capital investment [50,52]. UAE and MAE shorten extraction time, but uneven acoustic or electromagnetic field distribution during scale-up may affect reproducibility. Green-solvent systems such as DES and ILs show promising selectivity but face difficulties in solvent recovery and product separation [67,71,75]. Overall, no single technique simultaneously achieves high yield, high purity, and low solvent consumption.

Current research still has notable shortcomings. Most extraction studies focus primarily on CBD yield, whereas the composition and content of co-extracted impurities such as waxes, chlorophyll, and polyphenols are less frequently reported. In addition, the compatibility of different extraction methods with downstream purification steps such as winterization, chromatography, and crystallization has not been systematically compared, and the influence of extraction selectivity on downstream purification load remains poorly quantified. Therefore, the available data are still insufficient to determine which extraction method is most suitable for producing high-purity CBD.

Based on the available laboratory data, process selection should be guided by the target product (high-purity CBD) and downstream purification requirements. SFE tends to be preferred when high-purity CBD is required and capital investment is available. Ethanol maceration is often used for full-spectrum hemp oil, but for high-purity CBD, it may increase the impurity load and require more rigorous downstream purification. DES and ILs show potential as green alternatives, but their application to high-purity CBD production still requires evaluation of solvent recovery, product separation, and toxicity. Field-assisted extraction is generally suited to small-batch, high-value-added production. These recommendations should be regarded as application-dependent trends rather than universal rules, and industrial feasibility requires further validation.

Looking forward, CBD-extraction research is evolving from single-unit optimization toward integrated process design. Future work should move beyond yield-oriented evaluation and establish multi-dimensional criteria covering product quality, impurity loading, energy consumption, and economic performance. Coupled modeling of extraction and purification could enable coordinated optimization. Standardized reporting of extraction time, recovery, solvent consumption, and scale is also needed to make cross-process comparisons more reliable. Bridging the gap between laboratory findings and practical application will require further pilot-scale validation.

## 4. Separation and Purification Technologies

Crude CBD extracts are complex mixtures containing waxes, pigments, and THC alongside target cannabinoids. Regulatory limits on THC vary by country, and the heat-labile nature of CBD further imposes tight constraints on purification.

The impurity profile depends strongly on the upstream extraction method, and no single technique can adequately remove both non-specific impurities and THC while preserving CBD integrity. The main purification approaches are molecular distillation, chromatography, and crystallization, which are therefore typically used in combination to achieve purity targets for different product grades.

### 4.1. Winterization

Winterization is a conditional dewaxing pretreatment in downstream purification that removes waxes and lipids via low-temperature precipitation. It is typically positioned after crude extraction and before molecular distillation, and its necessity depends on the wax co-extraction tendency of the upstream process. Extracts obtained with hydrocarbon solvents, supercritical CO_2_, and room-temperature ethanol often require winterization, whereas low-temperature ethanol extraction reduces wax entrainment and may simplify or even eliminate this step.

Calmanti et al. [79] used a scCO_2_ extract containing 30.8% *w*/*w* CBD as feedstock and performed winterization in an acetonitrile system at −18 °C for 48 h. After scaling up to 100 g of feedstock, 54.7 g of dewaxed extract containing 48.5% *w*/*w* CBD and 45.3 g of wax residue containing 9.2% *w*/*w* CBD were obtained. Consistent results from the 20 g small-scale experiments confirmed the scalability of this protocol.

Upstream extraction temperature can effectively regulate the wax loading of crude extracts. Sosa et al. [80] demonstrated that lowering the butane extraction temperature from −9 °C to −46 °C reduced C_22_-C_31_ long-chain alkane waxes by more than 3-fold. The cannabinoid and aroma profiles remained nearly unchanged, which substantially alleviated the downstream winterization burden.

In summary, winterization should be optimized according to the wax co-extraction characteristics of the upstream extraction process, rather than being treated as a fixed and universal pretreatment step.

### 4.2. Molecular Distillation

Molecular distillation operates under high-vacuum conditions (0.1–10 Pa). It features short-term thermal exposure that mitigates thermal degradation of cannabinoids, and is widely adopted as a pretreatment unit for refining crude CBD extracts [81,82].

Molecular distillation equipment is classified into wiped-film and centrifugal types according to configuration. Wiped-film molecular distillation stands out for its continuous-operation mode, which exhibits prominent advantages when dealing with high-flow-rate and high-viscosity crude extracts, making it the dominant industrial-scale equipment. According to response-surface-methodology-based optimization, two-stage wiped-film distillation achieves efficient purification under optimal conditions of 40 Pa, evaporation temperature 170 °C and condensation temperature 20 °C, yielding CBD recovery of 92.66% and product purity of 80.19%. Operating pressure, evaporation temperature, condensation temperature and feed rate serve as key parameters governing cannabinoid recovery [83].

In contrast, short-path distillation mostly refers to laboratory-scale batch devices. When processing crude extracts containing 3.6 wt.% CBD and 0.2 wt.% THC at 1.33 kPa, it delivers around 97% CBD recovery and a product purity close to 90%. Restricted by batch-mode operation, it is only suitable for lab-scale process exploration [84].

Overall, molecular distillation offers favorable CBD enrichment capacity yet suffers from intrinsic limitations in separation selectivity. Owing to highly similar volatility between CBD and THC, complete separation cannot be achieved by distillation alone. This technology mainly removes volatile impurities such as terpenes, waxes and pigments, and functions merely as a pretreatment unit in purification workflows. Coupling with highly selective techniques such as preparative chromatography or crystallization is therefore indispensable to meet the purity and regulatory requirements for final CBD products [18]. These requirements vary according to the intended product category (e.g., food or pharmaceutical use) and the applicable jurisdiction.

### 4.3. Chromatographic Separation

Chromatography exhibits higher selectivity than molecular distillation and addresses the inherent limitation of molecular distillation in separating CBD from THC, rendering it a key polishing step for high-purity CBD. Based on stationary phase type and separation mechanism, chromatographic techniques can be grouped into three classes: liquid–solid, bonded-phase, and liquid–liquid partition chromatography. Continuous chromatographic approaches, such as simulated moving-bed chromatography (SMB) and recycling chromatography, do not change the underlying separation mechanism; they merely modify the operational mode to enhance separation efficiency.

#### 4.3.1. Liquid–Solid Chromatography

Liquid–solid chromatography employs solid-phase media and achieves fractionation via interfacial effects between analytes and stationary phases. Adsorption-and coordination-based chromatography realize selective separation through specific interfacial interactions, whereas size-exclusion chromatography performs fractionation via molecular-sieving effects with negligible adsorption under ideal conditions. This subsection summarizes the application characteristics and technical limitations of the three liquid–solid chromatographic approaches for CBD purification.

(1) Physical Adsorption Chromatography

Stationary phases based on physisorption mainly consist of macroporous adsorption resins and bare silica gel. Their separation performance is governed by specific surface area, pore-size distribution and elution conditions, yet they serve distinct roles in CBD-purification workflows.

Macroporous adsorption resins feature large adsorption capacity, adjustable selectivity and easy regeneration, and are widely used for industrial pre-enrichment of crude CBD extracts. These porous polymeric media selectively adsorb weakly polar CBD through van der Waals forces and hydrophobic interactions. Among available materials, non-polar HPD-100 resin (specific surface area: 650–700 m^2^/g, average pore diameter: 8.5–9.0 nm) exhibits balanced overall adsorption–desorption behavior. Under optimized conditions (pH 3.0, sample loading 2.6 BV, adsorption–desorption flow rate 2 BV/h, gradient ethanol elution), a 4.2-fold enrichment and an 83.13% recovery can be achieved, providing high-quality feedstock for subsequent polishing steps. An acidic pH of 3.0 maintains CBD in its non-ionized form, which favors resin adsorption kinetics. Combined with gradient ethanol elution for selective desorption, these factors jointly improve enrichment performance and recovery efficiency [85].

In a separate comparative study, six macroporous resins (DM-130, AB-8, D101, XAD-2, NKA-2, and H103) were evaluated for CBD enrichment. DM-130 gave the highest recovery (81.46%), while H103 gave the lowest (42.34%), with the remaining resins ranging from 59.13% to 72.34%. After enrichment with DM-130, the CBD purity in the dried product reached 28.93% [86]. This moderate purity indicates that macroporous resins are effective for initial enrichment but require further polishing to obtain high-purity CBD [86].

Bare-silica-gel normal-phase adsorption column chromatography represents a common laboratory-scale approach for preparing relatively high-purity CBD. Separation is achieved via hydrogen-bond-mediated physisorption by free silanol groups on particle surfaces, offering excellent separation resolution [87]. Nevertheless, this technique has inherent limitations, including irreversible adsorption of acidic cannabinoids such as CBDA, high organic-solvent consumption and limited scalability. For crude extracts obtained from industrial-hemp distillation residues, dry loading with n-hexane/ethyl acetate gradient elution affords 12.4 mg/g CBD per gram dry residue after column-chromatographic purification. As an alternative, Kugelrohr short-path distillation achieves a purification yield of 10.1 mg/g, while greatly reducing solvent consumption and improving scale-up potential [88]. Despite its low equipment cost, chromatography using bare silica gel suffers from severe scale-up restrictions and cannot be directly adopted as a core industrial-scale purification unit.

Overall, macroporous resins are characterized by high throughput and low operating costs, making them suitable for industrial pre-enrichment of crude CBD extracts. Bare-silica-gel normal-phase adsorption column chromatography provides superior resolution and is better suited for small-batch production of high-purity CBD samples. In practical industrial settings, these two techniques are not competitive alternatives but rather complementary unit operations that can be integrated to achieve both high throughput and high purity in CBD purification.

(2) Silver-Ion Coordination Chromatography

Compared with THC, CBD possesses a 1,5-diene skeleton offering two accessible olefinic sites, which is hypothesized to account for its stronger Ag^+^ affinity and enables effective differentiation of the two cannabinoids in Ag^+^-based separation systems [89].

Several studies have adopted an argentation strategy combined with ion-mobility spectrometry (IMS) and tandem mass spectrometry to distinguish cannabinoid isomers such as THC and CBD [90]. Silver-impregnated paper-spray mass spectrometry (PS-MS) also permits rapid discrimination and semi-quantitative analysis of these two cannabinoids [91].

Nevertheless, existing work remains largely confined to the analytical scale, predominantly relying on techniques such as IMS and PS-MS. Further studies are needed before industrial application.

(3) Size-exclusion chromatography

Size-exclusion chromatography (SEC) separates analytes according to differences in their hydrodynamic volume in solution. Porous gel media including Sephadex LH-20 are commonly used as stationary phases [11]. In cannabinoid purification, SEC functions primarily as a pre-purification step. It removes high-molecular-weight impurities such as chlorophyll, lipids, and waxes from crude hemp extracts, lowering sample complexity for subsequent fine-purification operations.

SEC can be coupled with preparative high-performance liquid chromatography (Prep-HPLC) to build a two-step “SEC-Prep-HPLC” purification workflow [92]. Crude hemp extracts undergo isocratic elution on Sephadex LH-20 with methanol as the mobile phase, and target fractions are collected. Fine separation is then carried out via Prep-HPLC using a water-acetonitrile gradient elution. This coupled process efficiently eliminates high-molecular-weight interferents, mitigates preparative-column fouling and stationary-phase degradation, and improves final-product purity.

Notably, CBD and THC possess identical molecular weights, so SEC cannot achieve their direct separation. Therefore, the merit of SEC in CBD-purification workflows does not reside in the final fractionation of target components. Instead, it acts as an efficient pre-treatment tool to simplify complex sample matrices, protect sophisticated chromatographic hardware, and boost the overall purification throughput.

#### 4.3.2. Bonded-Phase Chromatography

Bonded-phase chromatography employs organic functional groups covalently bonded to silica. Separation is governed primarily by partition, with secondary adsorption also affecting selectivity. C18 reversed-phase is the most common packing for CBD purification, whereas bonded normal-phase (cyano, diol, amino) provides complementary selectivity. This subsection summarizes their application characteristics and limitations for CBD purification.

(1) Bonded-phase C18 reversed-phase chromatography

In the field of preparative CBD purification, C18 reversed-phase chromatography stands as the most widely used separation medium. This stationary phase separates cannabinoids through hydrophobic interactions with its bonded octadecyl alkyl chains. The more lipophilic a cannabinoid molecule is, the stronger its hydrophobic association with the C18 alkyl chains, and thus the longer its retention time. CBD and THC share very similar structures but differ slightly in lipophilicity. This subtle hydrophobicity difference provides the molecular basis for their separation on C18 systems and has driven the iterative development of various process configurations [93,94].

Marzorati et al. [93] employed C18 flash chromatography as a downstream polishing step. After winterization of a supercritical CO_2_ extract containing 50.2% CBD, they processed 0.7 g of crude material per run. The C18 step could remove the psychoactive component THC and yield a CBD product with approximately 79% purity. Nevertheless, the separation power of a single flash column proved insufficient for reaching higher purity levels.

Ardakani et al. [94] took a further step by integrating semi-preparative C18 as the second-dimension polishing module in an offline two-dimensional liquid chromatography system, with CM2100 macroporous resin as the first dimension. The resin step provided an initial enrichment, yielding an intermediate CBD fraction at 84.1% purity. The subsequent C18 separation then removed remaining impurities through hydrophobic selectivity differences, elevating the final CBD purity to 94.3% with an overall recovery of 82.1%. This strategy substantially improved purity, but it required offline fraction collection and transfer between dimensions. From an industrial perspective, offline 2D-LC involves intermediate fraction storage, re-dilution and re-injection. These operations not only extend processing time but also introduce batch-to-batch consistency risks.

Overall, these C18-based strategies remain largely confined to the laboratory semi-preparative scale, with column diameters typically ≤10 mm and injection loads ranging from hundreds of milligrams up to ~1 g per run. When scaling up to industrial production, axial dispersion becomes more pronounced, and the trade-off between throughput and resolution emerges as a primary bottleneck. This challenge has shifted research focus from improving column efficiency alone toward optimizing system architecture. However, further gains from stationary-phase modification alone are approaching a plateau. Future advances are more likely to come from innovations in operating modes, such as continuous and recycling chromatography.

(2) Bonded-phase normal-phase chromatography

In normal-phase cannabinoid separation, silica gel bonded with cyano (CN), diol (Diol), or amino (NH_2_) groups represents the most widely used stationary phases. Compared with bare silica, these bonded-phase sorbents reduce non-specific adsorption via covalently grafted polar functional groups, delivering more uniform surface properties and faster solvent equilibration, thus overcoming bare-silica drawbacks such as unstable silanol activity and poor reproducibility.

De Luca et al. [95] systematically compared the retention behavior of neutral cannabinoids on various stationary phases. Under identical mobile-phase conditions, retention strength followed the order: amino-bonded phase > diol-bonded phase > cyano-bonded phase > bare silica. The elution order of cannabinoids was nearly reversed relative to C18 reversed-phase chromatography. From these observations, hydrogen-bonding interactions are inferred to be the key factor governing retention and selectivity within this system. Alcohol-modifier type exerted a significant influence on separation performance: under comparable conditions, ethanol yielded stronger cannabinoid retention than isopropanol, whereas the choice between n-hexane and n-heptane as the alkane component exerted negligible influence. Among the stationary phases tested, amino-bonded silica afforded the best overall resolution for THC, CBD, CBG, CBN, and CBC.

Compared with C18 reversed-phase systems, bonded-phase normal-phase chromatography offers several advantages for preparative workflows. It suppresses precipitation of terpenes and waxes originating from crude extracts, improving sample solubility and enabling higher loading capacities. Low-viscosity mobile phases generate limited backpressure, and solvent removal from collected fractions is also more straightforward. Nevertheless, system selectivity is highly sensitive to modifier composition; even trace water can induce retention-time drift. Furthermore, most existing investigations remain limited to analytical-scale condition screening. Scaling-up rules from analytical to preparative scales remain poorly understood, and the practical potential of continuous chromatographic processes within this configuration requires further exploration.

#### 4.3.3. Liquid–Liquid Partition Chromatography

Liquid–liquid partition chromatography (LLC) is a preparative separation technique in which the two phases of a biphasic solvent system serve as the stationary and mobile phases. It requires no solid support, thereby avoiding irreversible sample loss caused by solid-phase adsorption and making it particularly suitable for thermolabile natural products. LLC mainly includes centrifugal partition chromatography (CPC) and countercurrent chromatography (CCC).

(1) Centrifugal partition chromatography

As a typical solid-free liquid–liquid chromatographic technique, centrifugal partition chromatography (CPC) offers technical advantages and shows potential for industrial-scale application in the fine purification of cannabinoids [96,97]. Compared with conventional solid-phase chromatography, CPC completely avoids irreversible sample loss originating from stationary-phase adsorption, making it well-suited for large-scale preparation of heat-sensitive cannabinoids.

In the field of CBD purification, the core application value of CPC lies in precise THC removal for manufacturing compliant broad-spectrum CBD products. Studies have coupled low-temperature ethanol extraction with CPC polishing to achieve efficient differentiation between CBD and THC. After systematic solvent screening, the composite system of n-hexane/0.1% formic acid-acetonitrile/20 mmol/L ammonium formate delivers optimal separation performance with preferential elution of THC. After a single CPC run, the THC content in the product is reduced to below 0.2%, with a THC removal rate of 92%. A broad-spectrum CBD product with a purity of 73.3% and a recovery of 89.3% can be obtained. The process supports a sample loading of 5 g per run. Separation parameters can be linearly scaled according to rotor volume and flow rate, demonstrating favorable feasibility for industrial transfer [45]. In addition to conventional CPC operation, fast CPC (FCPC) configurations have also been validated for this purpose. Maly et al. [98] demonstrated that FCPC enables effective isolation of both CBD and CBDA from hemp extracts while successfully eliminating psychotropic phytocannabinoids, further confirming the versatility of liquid–liquid chromatographic technologies for cannabinoid refining.

pH-zone-refining CPC shows unique adaptability for acidic cannabinoids that cannot be efficiently separated by conventional workflows. By establishing two-phase solvent environments with distinct pH values, the partition-coefficient differences in acidic analytes can be greatly amplified, enabling targeted enrichment and focused elution of CBDA and related acidic cannabinoids. Using the composite biphasic system of n-hexane/ethyl acetate/ethanol/water with precise pH adjustment for both phases, followed by liquid–liquid extraction, CBDA with purity higher than 95% and overall recovery above 45% can be attained [99]. This approach eliminates the requirement for pre-separation of acidic fractions, simplifying pretreatment workflows and improving overall purification efficiency.

Against the background of green-separation-process upgrading, bio-based solvent systems are gradually replacing petroleum-derived solvents for cannabinoid purification by CPC. Low-toxicity bio-solvents, including hexamethyldisiloxane, ethyl isobutyrate and 2-methyltetrahydrofuran, can form stable biphasic systems with ethanol-water. While maintaining final CBD purity above 95%, such systems greatly reduce the environmental footprint of separation procedures and offer new technical routes for developing low-toxicity, recyclable and sustainable CBD polishing processes [100].

Overall, CPC remedies the engineering drawbacks of solid-phase chromatography such as adsorptive sample loss and limited loading capacity. It combines high separation resolution, high throughput and scale-up capability, and is applicable for differentiated purification of both neutral CBD and acidic cannabinoids. Nevertheless, its process stability is highly sensitive to solvent composition and pH-control accuracy. Standardized parameter sets for continuous industrial-scale operation remain insufficient. Future efforts should focus on solvent recycling, process-parameter standardization and continuous-equipment adaptation to further promote the industrial implementation of CPC for large-scale CBD purification.

(2) Countercurrent Chromatography

Countercurrent chromatography (CCC) is a class of solid-support-free liquid–liquid partition chromatography, with high-speed countercurrent chromatography (HSCCC) being its most representative implementation. HSCCC eliminates irreversible sample adsorption losses caused by solid-phase supports and shows great potential for preparative-scale isolation of bioactive constituents from plant-derived crude extracts [101]. Unlike CPC, which retains the liquid stationary phase via hydrostatic effects, HSCCC relies on hydrodynamic forces to retain the stationary-phase liquid inside the coiled tube. Satisfactory separation resolution can be achieved under laboratory-scale conditions; however, stationary-phase retention is highly sensitive to solvent density differences and rotor operating parameters, posing greater challenges for process reproducibility and practical scale-up compared with CPC [102].

In cannabinoid separation, HSCCC has shown promising application prospects. For example, patent US2022/0152523A1 employed a hexane/ethyl acetate/methanol/water (6:1:6:1, *v*/*v*/*v*/*v*) biphasic system and achieved effective separation of THC and CBD standards at the laboratory scale (injection amount approximately 2 mg) [103]. Ohtsuki et al. [104] applied countercurrent separation to a commercial “CBD oil” and, in addition to obtaining high-purity CBD, also isolated a more polar cannabinoid fraction containing cannabigerol (CBG) and cannabidivarin (CBDV), as well as a less polar fraction containing cannabichromene (CBC), trans/cis-Δ^9^-THC, and cannabinol (CBN). Furthermore, Luca et al. [105] used an HPCCC-Mini Centrifuge (CCC mode) for LLC experiments on pesticide removal from hemp extracts, further expanding the application scope of CCC in cannabinoid purification and remediation.

The main advantages of HSCCC include the absence of irreversible adsorption, high sample recovery, suitability for thermolabile compounds, and solvent recyclability, aligning with green separation requirements. However, its stationary-phase retention is highly sensitive to solvent density differences and rotor operating parameters, and stationary-phase loss tends to occur during scale-up, directly impairing separation resolution and reproducibility. Currently, most related studies remain confined to small-batch laboratory tests, and systematic engineering research on standardized process parameters for industrial-grade feedstock, mechanical optimization, and continuous supporting systems is still insufficient, constituting a major bottleneck for its translation toward industrial-scale cannabinoid manufacturing.

Overall, both CPC and CCC belong to LLC and share the advantages of no solid-phase adsorption, high sample recovery, and suitability for thermolabile cannabinoids. CPC relies on hydrostatic forces to retain the stationary phase, offering relatively stable retention and better feasibility for industrial scale-up. CCC/HSCCC relies on hydrodynamic forces and can achieve high resolution at the laboratory scale, but its stationary-phase retention is sensitive to solvent density and rotor parameters, making scale-up more challenging. The two techniques are complementary for cannabinoid purification, and common issues such as solvent selection, process standardization, and continuous operation remain key challenges for further industrial application.

#### 4.3.4. Continuous Chromatography

Conventional batch chromatography is widely used for laboratory-scale CBD separation; however, its long separation cycles, poor batch-to-batch reproducibility, high solvent consumption, and discontinuous operation limit its industrial scale-up. Continuous chromatography can significantly improve throughput and solvent efficiency, offering considerable potential for the industrial-scale purification of high-purity CBD. This subsection compares the performance, applicability, and scale-up limitations of simulated moving-bed chromatography (SMB) and recycling chromatography.

(1) Simulated moving-bed continuous chromatography

As a continuous chromatographic technique with considerable industrial-application potential, SMB adopts a multi-column configuration combined with programmed-valve switching to mimic solid–liquid counter-current contact, enabling continuous sample feeding and on-line fraction collection [106]. It delivers effective resolution for structurally closely related cannabinoids such as CBD and THC.

An eight-column reversed-phase SMB setup has been investigated for continuous THC depletion from cannabis extract using the eco-friendly ethanol-water mobile phase (80% ethanol/20% water). The system was equipped with eight columns (250 × 8.0 mm, 15 μm C18 fully porous particles, column volume 12.56 mL each) and operated with a feed flow rate of 0.08 mL/min using a CBD-rich ethanolic extract (initial CBD concentration 11.7 mg/mL, initial purity 88%). Under optimized operating conditions, CBD with ca. 98% purity and 99% recovery was obtained. The SMB process achieves a solvent consumption of merely 3.29 L/g CBD, representing over 90% reduction compared with 41.05 L/g CBD of batch chromatography. In terms of productivity, SMB yields 0.575 g/(h·L) (based on total column volume) and an absolute productivity of 56.8 mg/h (based on the 8-column system with a total column volume of 100.5 mL), approximately 10-fold higher than that of batch workflows (5.85 mg/h) [107].

Benefiting from continuous-operation mode and green ethanol-water elution system, SMBC demonstrates promising potential for continuous CBD production with high purity and recovery. However, current validation is primarily at the laboratory/preparative scale, and future pilot-scale studies are required to confirm its feasibility for industrial manufacture. Nevertheless, the process requires precise multi-column parameter matching and valve-timing control. Waxes and pigments in crude extracts may trigger stationary-phase fouling and impair long-term system stability. Pre-purification is thus required for complex feedstock to exert the full continuous-production merits of SMB.

(2) Recycling chromatography

Recycling chromatography improves resolution by repeatedly passing unresolved fractions through the column, which is equivalent to extending the effective column length. This operating mode does not change the separation mechanism but amplifies the resolution gain from the stationary phase.

Compagnin et al. [108] applied this concept to CBD purification using a twin-column recycling chromatography setup with two 150 × 8 mm semi-preparative C18 columns. By switching valves, the sample circulated repeatedly between the two columns. Three valve switches delivered a separation performance equivalent to that of five columns in series. Using 100% ethanol as the mobile phase and eliminating intermediate fraction transfer, they obtained CBD at 97.3% purity with a recovery of 99.7%, and no THC was detectable in the target fraction.

However, these performance metrics were achieved under conditions of very low loading (20 μL injection volume) and extended cycle times (multiple column passages). Compared with conventional single-column preparative chromatography, the throughput per unit time of recycling chromatography is substantially lower, which poses economic challenges for large-scale CBD API production. Therefore, recycling chromatography is better suited for difficult separations or small-scale high-purity production, rather than as a core industrial-scale purification unit. Realizing continuous operation while maintaining high resolution has thus become a central focus in recent process development.

(3) Comparison and scale-up considerations

SMB provides high throughput and low solvent consumption, making it suitable for continuous industrial-scale production. In contrast, recycling chromatography offers higher resolution but lower throughput, making it more appropriate for difficult separations or small-scale high-purity production. These two continuous modes are complementary rather than mutually exclusive, and their selection depends on the required scale, feed complexity, and target purity.

### 4.4. Purification by Crystallization

Crystallization is the mainstream refining technology for producing industrial-grade high-purity CBD crystals, relying on component-based solubility differences for separation. Compared with preparative chromatography and molecular distillation, this technique features moderate capital investment and recyclable solvents, rendering it suitable for large-scale manufacture. Rational design of crystallization processes depends on thermodynamic solubility data and fundamental crystallization-kinetics knowledge. Translating such fundamental understanding into industrially scalable protocols remains a key challenge bridging laboratory research and practical applications.

#### 4.4.1. Batch Cooling Crystallization

CBD-enriched fractions are dissolved in alkanes or alcohols to prepare near-saturated solutions. CBD precipitates upon programmed cooling and seeding, while impurities remain in the mother liquor. Pulido-Bonilla et al. [109] measured CBD solubility in n-pentane, n-hexane, n-heptane and isooctane across 253.1 K–310.1 K via the isothermal method. DSC delivered key thermodynamic parameters: melting point of 341.4 ± 0.4 K and fusion enthalpy of 21 ± 3 kJ/mol. These results support solvent screening and the definition of operable crystallization temperature windows.

On this basis, Pulido-Bonilla et al. [110] carried out batch cooling-crystallization experiments using n-heptane as a solvent. The effects of crystallization temperature, solvent-to-CBD-enriched-fraction mass ratio, and stirring speed were investigated. Temperature and solvent-to-enriched-fraction ratio were confirmed as dominant factors governing product purity and yield, whereas stirring speed showed negligible influence. At a solvent-to-CBD-enriched-fraction mass ratio of 0.5:1 and −10 °C, white needle-to-rod-shaped CBD crystals were obtained with 99.997% purity and 71.98% yield. Washing with cold n-heptane increased product purity by 4.16% on average. Drying barely improved purity (ca. 0.063%), yet effectively removed residual solvent and suppressed crystal agglomeration, which is critical for downstream powder-processing performance. XRD characterization verified that under these conventional batch cooling conditions, batch cooling crystallization exclusively yields thermodynamically stable Form I with a monoclinic crystal system. Impurity lattice inclusion is frequently observed, triggering crystal agglomeration, unit-cell parameter shift, and yellow discoloration.

CBD exhibits polymorphic behavior. Under the standard operating conditions described above, only stable Form I forms under conventional cooling crystallization with self-seeding. In contrast, Straker et al. [111] employed a non-routine approach: they adopted CBD analogs as template seeds and obtained metastable Form II through high-throughput laboratory crystallization, which is not a typical industrial operation. Form II exhibits a low melting point of 43–46 °C and poor thermal stability, and it readily transforms spontaneously into Form I. Although this metastable polymorph does not change chemically measured CBD content, it alters crystal morphology, powder flowability and dissolution performance. As reviewed by Filer C N. [112], Form I dominates industrial crystallization operations, and metastable Form II is unlikely to form under normal operating conditions. Even so, risks originating from abnormal processing conditions should not be overlooked; powder X-ray diffraction is recommended for routine polymorph quality control.

#### 4.4.2. Separation of THC by Crystallization

THC is a critical impurity requiring strict control during CBD purification. It is challenging to reduce THC below regulatory thresholds by chromatography alone. Crystallization realizes selective separation based on distinct solubility behaviors in weakly polar solvents: CBD preferentially crystallizes during cooling, while THC largely remains in the mother liquor owing to its poor crystallization tendency. It should be noted that crystallization alone cannot fully eliminate THC.

Calmanti et al. [79] used industrial-grade cannabis inflorescence containing approximately 4.8% CBD as feedstock and built a scalable integrated workflow consisting of thermal decarboxylation, supercritical CO_2_ extraction, acetonitrile winterization, C18 reversed-phase silica filtration, silica-gel column enrichment and subsequent selective crystallization to retain THC in the mother liquor. At a 100–600 g preparation scale, the overall CBD yield reached 52%, product purity exceeded 99%, and THC content was negligible (<0.2%). Coupling crystallization with chromatography or molecular distillation enables production of high-purity CBD crystals with low THC residues without substantial yield sacrifice, representing a vital technical route for high-purity CBD manufacture.

In summary, most published experimental investigations focus on batch cooling crystallization, and reliable pilot-scale amplification data remain scarce. Multi-stage continuous crystallization and antisolvent crystallization have been proposed in patent literature; nevertheless, publicly available process parameters and validation data are limited. From an application-oriented perspective, future research should prioritize the following directions. First, predictive design strategies for crystallization processes should be established based on multi-component solid–liquid equilibrium thermodynamic models to reduce empirical trial-and-error work. Second, GRAS-listed solvents such as ethanol and ethyl acetate should be explored to replace n-heptane and alleviate safety concerns regarding residual solvents in pharmaceutical-grade end-products. Third, integrated continuous-industrial chromatography or membrane separation technologies can be deployed to recover residual CBD from mother liquors for higher overall yield. Further research in the above-mentioned aspects will promote CBD crystallization technology from laboratory-scale exploration toward efficient, controllable and compliant industrial manufacture.

## 5. Process Trade-Offs and Integration of Purification Technologies

Following the discussion of extraction and purification technologies, this section addresses process-level trade-offs and integration. It first provides a quantitative comparison of core purification techniques, then proposes a staged-integrated purification route with defined stage-wise functions, compares the applicability of different process routes, and finally presents a critical assessment of industrial scale-up challenges, with particular emphasis on continuous chromatography. The optimal combination is shown to depend on the target product, production scale, feed purity, and applicable regulatory requirements.

### 5.1. Quantitative Comparison of Core Purification Techniques

After extraction, the purity and quality of the final CBD product are mainly determined by the downstream purification steps [11,12]. However, these steps differ widely in separation mechanism, operating conditions, and industrial maturity, making direct comparison difficult [13,14]. Reported purity and recovery values are often obtained under different feedstock compositions, initial CBD contents, and analytical methods, and many studies do not report solvent consumption, throughput, or scale [13,43]. A systematic comparison is therefore necessary to identify the strengths and limitations of each technique and to provide a basis for process integration. Table 2 summarizes the available quantitative data for the main purification unit operations.

In terms of purity and recovery, molecular distillation provides moderate purity but high recovery, and its main limitation is the inability to separate CBD from THC, making it suitable mainly for pre-enrichment [83,84]. Batch chromatography shows highly variable performance depending on the mode: some modes are suitable only for crude enrichment [86], while others can reach high purity, but they generally suffer from high solvent consumption, low throughput, and scale-up challenges [45,94]. SMB achieves both high purity and high recovery under continuous operation [107]. Recycling chromatography also offers high purity and recovery, but its loading is very low and cycle times are relatively long, making it more suitable for difficult separations than for large-scale production [108]. Crystallization delivers the highest purity, but its recovery is moderate, it requires a high-purity feed, and it removes THC only partially [110].

In terms of solvent consumption and productivity, SMB consumes substantially less solvent than batch chromatography and achieves roughly tenfold higher productivity [107]. Batch chromatography is flexible but suffers from high solvent consumption and low throughput, with the C18 mode facing a particular throughput–cost bottleneck [94]. Recycling chromatography has very low loading and long cycle times, which limits its scalability [108]. Crystallization uses recyclable solvents and has moderate throughput, but its performance depends strongly on upstream purity [110]. Winterization is a conditional step that removes waxes only and does not separate THC, but it can reduce the load on downstream distillation and chromatography [13,79,93].

Overall, no single unit operation simultaneously satisfies high purity, high recovery, low solvent consumption, high throughput, low cost, and good scalability [11,12,14]. Molecular distillation is suitable for pre-enrichment, chromatography for selective separation, crystallization for final polishing, and winterization as a conditional pretreatment. These techniques are complementary rather than substitutes, and their integration is essential for high-purity CBD production [14,43]. Based on this understanding, the next section proposes a staged-integrated purification route, which serves as the basis for the route comparison and scale-up discussion that follow.

### 5.2. Proposed Integrated Purification Route and Stage-Wise Functions

**Figure 4:**
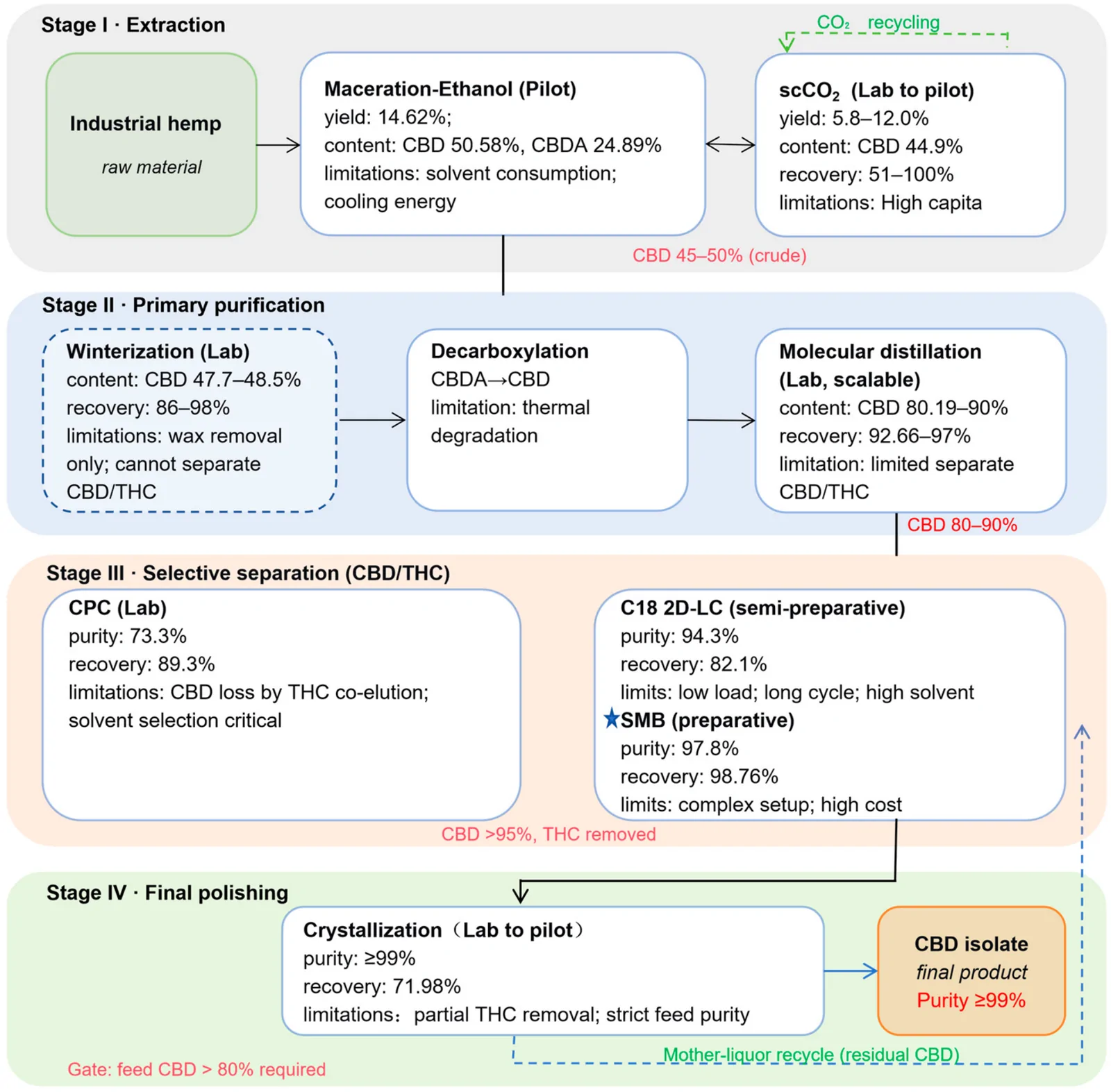
Integrated extraction and purification workflow for producing high-purity CBD from industrial hemp. The process includes extraction (ethanol/scCO_2_), primary purification (winterization, decarboxylation, and molecular distillation), selective separation (chromatography for CBD/THC separation), and final polishing (crystallization). Each step plays a distinct separation role. The four background colors indicate the distinct processing stages (Stage I to IV). Black solid arrows indicate the process flow, and the red text indicates the typical CBD purity specifications for each stage. Green dashed arrows indicate solvent or material recycling. The star highlights SMB as a promising continuous chromatographic strategy.

In practice, the overall process is better organized into functional stages, each addressing a specific separation task and preparing the feed for the next stage. This staged approach allows the process to balance purity, recovery, solvent consumption, and industrial feasibility. Based on this principle, a four-stage integrated route is proposed and illustrated in Figure 4.

Stage I (extraction) provides the crude CBD feed. Supercritical CO_2_ extraction typically yields extracts with lower wax and pigment content, whereas ethanol extraction co-extracts more waxes and colored compounds; this difference affects the load on winterization and chromatography [79]. Regardless of the extraction route, the subsequent purification tasks remain similar: removing bulk impurities, separating CBD from THC, and achieving final purity.

Stage II (primary purification) aims to remove bulk impurities and reduce the load on downstream chromatography. Winterization removes waxes and lipids, while thermal decarboxylation converts CBDA to CBD. Molecular distillation further separates volatile and heavy components under solvent-free conditions. This stage significantly increases CBD concentration in the feed, but it cannot separate CBD from THC. Therefore, primary purification should be regarded as a pre-enrichment step rather than a final purification step.

Stage III (selective separation) is responsible for resolving structurally similar cannabinoids, especially CBD and THC. Chromatographic techniques are central to this stage. Batch chromatography, including C18, CPC, and HSCCC, offers high resolution at laboratory and pilot scale, but faces challenges in solvent consumption, throughput, and stationary-phase stability [45,94,102]. Simulated moving bed (SMB) chromatography provides continuous operation, high purity, high recovery, and low solvent consumption, making it a promising candidate for future industrial-scale production, although it requires higher capital investment and precise process control [107]. The choice among these techniques depends on the target product, production scale, and THC limit required by the applicable regulatory framework [9].

Stage IV (final polishing) is typically achieved by crystallization. Crystallization can deliver CBD crystals with purity exceeding 99%, but it requires a high-purity feed, generally with CBD content above 80% [110]. It also removes THC only partially, so the upstream chromatographic step must already reduce THC to the required level. The mother liquor from crystallization can be recycled to recover residual CBD, improving the overall yield [79].

Across all stages, solvent recovery, online process monitoring, and mass balance control are essential for industrial feasibility [43]. The proposed route is not a fixed recipe but a functional framework: different feedstocks, product specifications, and regulatory requirements may require different combinations of these stages [9,13]. The key is to match each stage to its intended function, rather than to maximize the complexity of the process.

### 5.3. Comparison and Applicability Analysis of Process Routes

**Figure 5:**
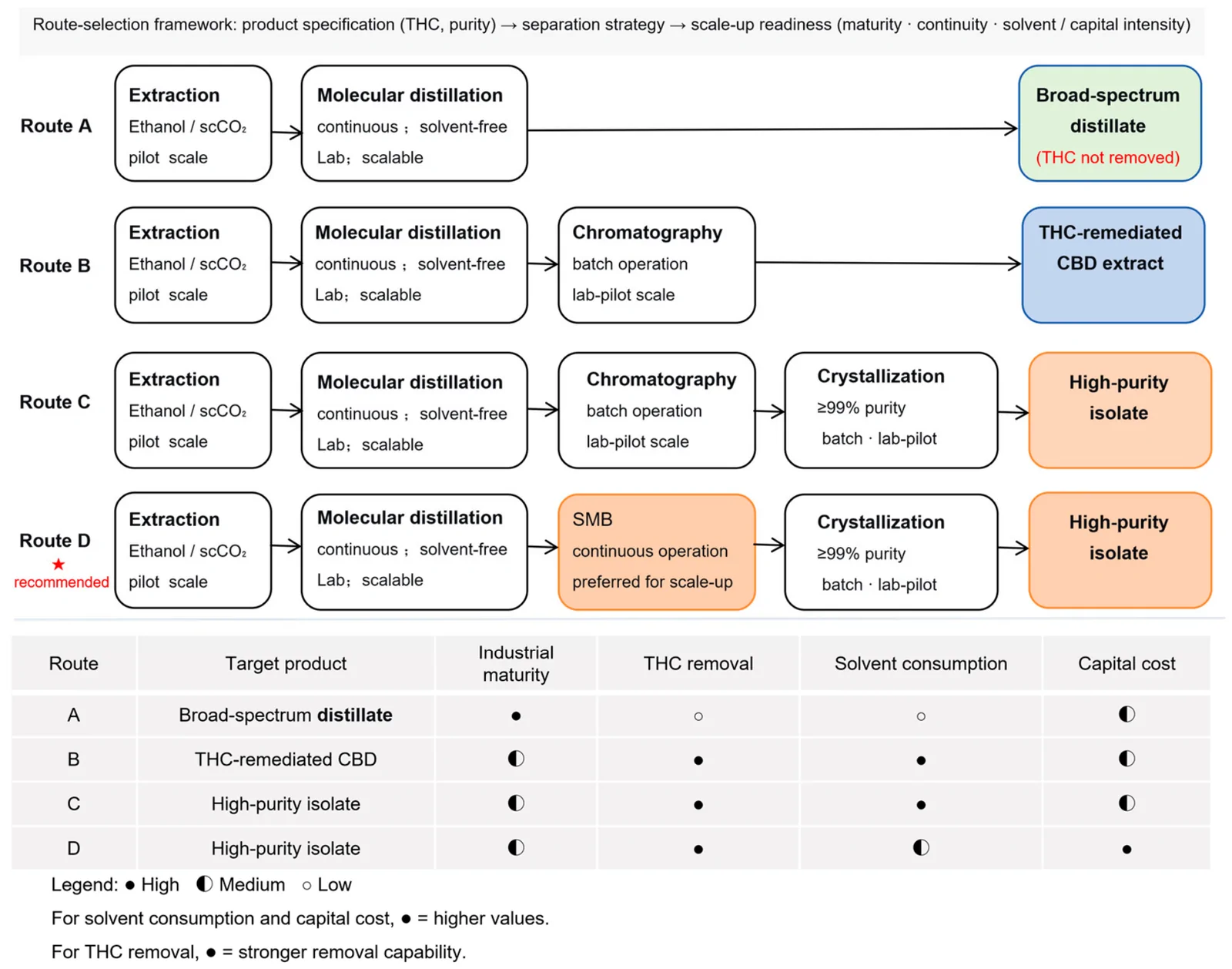
Industrial maturity: high—unit operations established at industrial or mature pilot scale (ethanol extraction, 3 kg → industrial; molecular distillation: continuous, solvent-free [83,84]); medium—lab–pilot demonstration (batch chromatography ≤ 5 g/run [45,94,103]; SMB lab/preparative [107]). SMB is currently at lab/preparative scale [107]; its continuous operation and order-of-magnitude higher productivity versus batch chromatography give it the highest scale-up readiness; Route D is highlighted as the preferred proposed conceptual route for future industrial translation. Route A has no THC-separation step (molecular distillation cannot separate CBD/THC [83,84]); THC compliance relies on feed selection and dilution.

The selection of an integrated purification route is not determined by the number of unit operations alone, but by the required balance among product purity, THC removal, solvent consumption, capital cost, and production scale [11,12,14]. These factors are interdependent: increasing purity usually requires additional separation steps, which in turn raise solvent consumption and capital investment, while continuous operation can reduce solvent use and improve productivity at the expense of higher technical complexity [107]. To illustrate these trade-offs, four representative routes (Routes A–D) are summarized in Figure 5 and briefly described below.

Route A (extraction + molecular distillation) is suitable when the target product is a broad-spectrum extract and THC content is not a critical constraint. Molecular distillation enables pre-enrichment under solvent-free conditions but cannot separate CBD from THC [83,84]. This route is cost-effective and simple, and is appropriate for products with moderate purity requirements.

Route B (extraction + molecular distillation + batch chromatography) is used when THC removal is necessary but ultra-high purity is not yet required. Batch chromatography, such as C18, CPC, or HSCCC, provides high resolution at laboratory and pilot scale, but suffers from high solvent consumption, low throughput, and scale-up challenges [45,94,102]. This route is suitable for small-to-medium-scale production where cost is sensitive but a specific THC limit must be met.

Route C (extraction + molecular distillation + chromatography + crystallization) offers a practical balance between purity and recovery for small-to-medium-scale high-purity CBD isolates. Crystallization can deliver purity exceeding 99%, but requires a high-purity feed (CBD > 80%) and removes THC only partially [110]. Therefore, the upstream chromatographic step must already reduce THC below the regulatory limit. This route has been validated at laboratory scale and scaled up to 100–600 g in a multi-step process [79].

Figure 5 Route-selection framework for producing high-purity CBD from industrial hemp. The colored boxes on the right (green, blue, and orange) represent the target products. The red label highlights Route D as the recommended option, and black solid arrows indicate the process flow. Industrial maturity: high denotes unit operations established at industrial or mature pilot scale (ethanol extraction; continuous molecular distillation [83,84]); medium denotes lab-to-pilot demonstrations (batch chromatography and SMB [45,94,103,107]). Although SMB is currently at the lab/preparative scale, its continuous operation and significantly higher productivity versus batch chromatography give it strong scale-up readiness. Therefore, Route D is proposed as the preferred conceptual route for future industrial translation. In contrast, Route A lacks a THC-separation step because molecular distillation cannot separate CBD/THC [83,84]; thus, THC compliance relies solely on feed selection and dilution.

Route D (extraction + molecular distillation + continuous SMB chromatography + crystallization) represents a proposed conceptual industrial route for the production of high-purity CBD. Replacing batch chromatography with continuous SMB chromatography substantially reduces solvent consumption and increases productivity (approximately 10-fold) [107]. SMB achieves a purity of 98% and recovery of 99% with a solvent consumption of 3.29 L/g. However, its industrial scale-up still needs to address stationary-phase lifetime, long-term stability, cleaning validation, and capital investment. This route is suitable for industrial products with strict requirements on purity, THC limit, and batch-to-batch consistency.

The choice among these routes depends on the target product category, feed purity, production scale, and applicable regulatory framework [9]. A broad-spectrum extract may only require Route A, whereas a high-purity CBD isolate requires Route C or D. Notably, the industrial feasibility of Route D depends on the scale-up maturity of continuous chromatography, which remains a key bottleneck in industrial scale-up [107]. In addition, THC limits vary among jurisdictions (e.g., <0.2%, <0.05%), directly affecting the number and stringency of required separation steps [9].

Although the above routes have been validated at laboratory and pilot scale, direct scale-up assessment remains limited. Most reported studies are at laboratory scale, and data on recovery, solvent consumption, throughput, and long-term stability are often incomplete [43]. Standardized process parameters, continuous-flow equipment, solvent-recycling infrastructure, and techno-economic/life-cycle assessments are still lacking [43,82,83,84]. Therefore, the next section provides a dedicated critical discussion of industrial scale-up challenges.

### 5.4. Industrial Scale-Up: Critical Assessment and Challenges

Although the extraction and purification techniques discussed above have been widely validated at laboratory and pilot scale, industrial-scale data for high-purity CBD production remain scarce [43]. This section provides a critical assessment of industrial scale-up from five perspectives: classification of scale-up bottlenecks, continuous chromatography scale-up, purity–recovery–cost trade-offs, techno-economic and life-cycle assessments, and regulatory and batch-consistency requirements.

#### 5.4.1. Classification of Scale-Up Bottlenecks

The bottlenecks in industrial scale-up can be grouped into four categories:

(1) Thermodynamic bottleneck

CBD and THC are structural isomers with similar retention behavior and low separation factors [11,12,95]. Therefore, thermodynamic selectivity alone is insufficient for complete separation, and multi-step coupling or highly selective separation media are required.

(2) Hydrodynamic bottleneck

In continuous chromatography, stationary-phase loss, mass-transfer limitations, and flow/pressure fluctuations during long-term operation are common issues; advanced process control is increasingly recognized as a prerequisite for stable preparative operation [106]. Although SMB has achieved 98% purity and 99% recovery at laboratory scale [107], its industrial scale-up must address stationary-phase lifetime, long-term stability, and cleaning validation.

(3) Engineering bottleneck

Supercritical CO_2_ extraction requires high capital investment and faces challenges in maintaining uniform mass transfer at large scale [47,48,50]. Notably, extraction scale-up has advanced faster than purification scale-up: industrial-scale microwave-assisted ethanol extraction has already been demonstrated [64], whereas chromatography still consumes large amounts of solvent and solvent-recovery infrastructure remains insufficient.

(4) Economic bottleneck

Continuous chromatography requires high equipment investment, and green solvents such as DES and ILs, although reducing volatility and toxicity, still suffer from high viscosity, difficult recovery, and limited recyclability, hindering large-scale application [68,69,73].

#### 5.4.2. Continuous Chromatography Scale-Up

SMB is currently the most industrially promising continuous chromatography technique. At laboratory/preparative scale it has achieved 98% purity, 99% recovery, productivity of 56.8 mg/h (≈10-fold that of batch chromatography), and solvent consumption of 3.29 L/g [107]. Importantly, the industrial feasibility of continuous chromatography is already indicated by patented processes in which SMB was applied to THC remediation at >99% purity and twin-column MCSGP configurations delivered CBD at >99.5% purity [11]. Nevertheless, the only peer-reviewed CBD-focused SMB study remains at laboratory/preparative scale, and industrial scale-up must verify stationary-phase lifetime and regeneration, long-term operational stability, cleaning and validation procedures, and capital and operating costs.

Beyond SMB and recycling chromatography, advanced liquid–liquid chromatography (LLC) operating modes have been systematically evaluated for the THC remediation of hemp extracts: batch elution provided good productivity and a THC-free extract, the semi-continuous trapping multiple dual mode showed lower productivity than batch, whereas the continuous sequential mode delivered a THC-free extract of similar composition with superior productivity, providing a model-based basis for industrial design. Recycling chromatography can achieve 97.3% purity and 99.7% recovery [108], but its loading is extremely low (20 μL injection), cycle time is long (more than 30 min/run), and solvent consumption is relatively high, making it more suitable for difficult separations than for large-scale production.

#### 5.4.3. Purity–Recovery–Cost Trade-Offs

There is an inherent trade-off among purity, recovery, throughput, and cost. Chromatography can achieve high purity but suffers from low throughput and high solvent consumption; crystallization can provide very high purity (≥99%, up to 99.997%), but its recovery is moderate (71.98%), it requires a high-purity feed (CBD > 80%) [110], and the entire extraction-to-crystal procedure has only been demonstrated at laboratory scale [79], with solvent selection governed by CBD solubility [109]. Molecular distillation and chromatography are energy-intensive, and continuous processes require higher initial investment. Therefore, no single technique can simultaneously satisfy all metrics, and industrial routes must be selected according to product positioning.

#### 5.4.4. Techno-Economic and Life-Cycle Assessments

TEA and LCA studies of the hemp/CBD value chain have so far concentrated on the upstream segments: techno-economic analyses have quantified the production cost of CBD-rich oil at 6.71–11.15 USD/kg [113], and life-cycle assessments have addressed the residual hemp biomass generated during CBD extraction—approximately 90% of harvested dry matter, amounting to roughly 82,000 t in the United States in 2019–2021 [114]. In contrast, comprehensive TEA and LCA targeting the purification train itself (chromatography–crystallization integration) are still lacking [43,82,83,84]. TEA should cover capital investment, operating costs, solvent recovery rates, and product yields; LCA should evaluate energy consumption, solvent use, and environmental impact. Without such assessments, industrial scale-up decisions lack a quantitative basis.

#### 5.4.5. Regulatory and Batch-Consistency Requirements

Industrial-scale production must meet batch-to-batch consistency, quality control, and regulatory requirements, which vary by jurisdiction and product category [6,9]. THC limits differ among countries (e.g., <0.3%, <0.2%, <0.05% *w*/*w*), directly affecting the number and stringency of required separation steps. The development of GMP-grade full-extract processes further illustrates the level of process standardization required for pharmaceutical-grade cannabinoid manufacturing [41]. Batch-to-batch feedstock variability (cultivar, cultivation conditions, harvest time, particle size) also affects extraction yield and impurity load [13,34,43], requiring standardized raw material specifications and on-line or at-line analytical monitoring.

Overall, industrial-scale production of high-purity CBD requires an integrated approach that considers feedstock variability, solvent recovery, continuous separation, purity–recovery trade-offs, energy consumption, and regulatory compliance. Currently, most studies remain at laboratory or pilot scale, industrial-scale purification data are scarce, and TEA/LCA studies specifically covering the purification train are still lacking. The large volume of residual biomass inherent to CBD production [114] further underlines the need for closed-loop solvent recycling and valorization of side streams. Addressing these gaps requires pilot-scale validation, continuous chromatography scale-up, solvent-recycling infrastructure, and purification-focused TEA/LCA to bridge the gap between laboratory research and industrial practice.

## 6. Summary and Outlook

Industrial-hemp-derived CBD preparation includes two core procedures: extraction and purification. Current industrial extraction is predominantly performed via supercritical CO_2_ extraction and ethanol solvent extraction. Supercritical CO_2_ extraction operates at low temperatures with zero solvent residue, making it suitable for high-quality CBD production, whereas it suffers from high capital investment. In comparison, ethanol extraction possesses mature technical routes and excellent scale-up feasibility, but it inevitably causes solvent residue issues and heavy post-desolvation burdens. Physical field-assisted techniques can be adopted to further improve mass transfer efficiency.

For the purification stage, molecular distillation, preparative chromatography, and crystallization are the dominant unit operations. Restricted by multiple trade-offs among purity, recovery, throughput and operational cost, individual purification techniques cannot satisfy comprehensive industrial requirements. Consequently, multi-process integration has become the mainstream industrial strategy for CBD manufacturing. Different integrated workflows can be designed to meet different purity levels and regulatory requirements. An integrated four-stage coupling route (extraction, primary purification, selective separation, and final polishing) has demonstrated the potential to achieve high purity and effective removal of trace THC at the laboratory scale, whereas simpler two-stage schemes (extraction + primary purification) may be more cost-effective when ultra-high purity is not required. Furthermore, single chromatographic purification is currently limited to laboratory-scale trials, and no universal process is optimal for all production scenarios.

Several bottlenecks still hinder the large-scale industrialization of CBD production. Most existing processes rely on discontinuous batch operation, resulting in poor procedural continuity. High equipment investment for supercritical systems and excessive solvent consumption during chromatographic purification substantially increase overall production costs. Moreover, batch-to-batch feedstock fluctuations degrade process stability, and low utilization efficiency of by-products (e.g., crystallization mother liquor) further restricts simultaneous improvement in yield, purity and economic benefit.

Future research can focus on five promising development directions:

(1) Construct modular multi-technique integrated systems, such as ultrasound-supercritical and enzyme-assisted ethanol extraction, to strengthen procedural connection and achieve process intensification.
(2) Develop highly specific separation media (e.g., molecularly imprinted polymers) combined with membrane separation technology to enhance target selectivity and reduce material consumption in purification procedures.
(3) Promote the industrial translation of continuous chromatographic techniques represented by simulated moving bed chromatography to promote the transformation from traditional batch operation to continuous production.
(4) Integrate online detection and intelligent algorithms to realize dynamic parameter regulation and improve process robustness against complex and variable feedstock matrices.
(5) Establish scenario-differentiated process systems and promote the recycling of crystallization mother liquor and extraction by-products to improve the sustainability of green CBD manufacturing.

In conclusion, the deep integration of multi-unit coupling, innovative separation techniques, continuous process upgrading, and intelligent control is the core pathway to break through the industrial bottlenecks of CBD purification and build efficient, stable and eco-friendly large-scale production systems.

## Figures and Tables

**Table 1:** Comprehensive comparison of four CBD extraction technologies for industrial hemp.

Extraction Category	Representative Processes	Extraction Time	Yield/Content/Recovery	Solvent Consumption	Reported Scale	Key Advantages	Key Limitations	References
Conventional organic-solvent extraction	Maceration/reflux	Lab: 21 °C, 30 min × 3; Pilot: −31 °C, 33 min	Lab: cannabinoid content 43.1% (pentane), 29.5% (diethyl ether), 20.3% (ethanol) [46]. Pilot: extract yield 14.62%, CBD 50.58%, CBDA 24.89%, THC 2.41% [45].	Ethanol; high; recovery required; lab 5:1 *v*/*w*; pilot 1.8% *w*/*v* (≈55.6 L/kg)	Lab scale; Pilot scale (3 kg)	Simple equipment; well-established; cold ethanol reduces chlorophyll/wax	Co-extracts polar impurities; high solvent consumption; cooling energy; THC still ~2.4%	[45,46]
Supercritical-fluid extraction	SFE (CO_2_)	Not specified (S/F 20–25)	Crude extract yield 5.8–12.0%; CBD 44.9% at 200 bar; total cannabinoid recovery 51–100%;	Solvent-free (CO_2_ recycled); 5% ethanol if used	Lab to pilot (0.5–10 L; 10 L pilot plant)	Low-temperature; low impurity; no solvent residue	High capital	[50]
Field-assisted extraction	UAE/MAE	UAE: tens of minutes; MAE: minutes to tens of minutes	UAE: 17.54% extract yield; 0.8752 mg/mL CBD [56]; MAE with 2-MeOx: 86.76 mg CBD/g dry matter [63]	UAE: ethanol or ethanol–water (e.g., 50% ethanol); MAE: 2-MeOx or ethanol; reduced vs. conventional	Lab to pilot	Enhanced mass transfer; shortened time	Field non-uniformity; hot-spot degradation	[56,63]
Emerging green-solvent extraction	DES/ILs	Tens of minutes to hours	DES: 11.07 mg CBD+CBDA/g hemp [67]; IL: 7.23 mg CBD/g hemp [71]	High	Lab scale	Low volatility; low toxicity potential	High viscosity; recovery and separation challenges	[67,71,75]

Table note: Reported recovery and yield values depend on feedstock, process conditions, and analytical methods, and should not be directly compared across studies. The “reported scale” indicates the experimental scale documented in the cited literature; most studies were conducted at the laboratory scale, and pilot-scale data remain scarce.

**Table 2:** Quantitative comparison of core purification techniques for CBD production from industrial hemp.

Unit Operation	Purity (CBD, % *w*/*w*)	Recovery (%)	Solvent System (Consumption)	Throughput/Productivity	Reported Scale	Key Limitations	References
Winterization	47.7–48.5	~86 (single pass); ~98 after repeated winterization	Acetonitrile (optimal) or ethanol (alternative); 3:1 *w*/*w* (3.8 mL/g)	20–100 g extract/batch	Lab to pilot scale (20–100 g)	Wax removal only; no THC separation	[13,79,93]
Molecular distillation	WFMD: 80.19 [83]; SPD: 89.4 [84]	WFMD: 92.66 [83]; SPD: 97 [84]	Solvent-free	WFMD: continuous, 100 mL/h [83]; SPD: batch, 500 mL/run [84]	Lab scale (2-inch WFMD [83]; 2 L glass flask [84])	Limited CBD/THC separation	[83,84]
Batch chromatography (DM-130)	28.93	81.46	n.r.	n.r.	Industrial pre-enrichment		[86]
C18 chromatography (2D-LC)	94.3	82.1	Ethanol/water; 75% EtOH (1st dim.), 91% EtOH (2nd dim.), isocratic	8.71–10.35 mg/h	Lab/semi-preparative scale	throughput–cost bottleneck	[94]
Batch chromatography (CPC)	73.3	89.3	n-Hexane/0.1% formic acid–acetonitrile/20 mM ammonium formate	5 g/run	Lab scale	CBD loss by THC co-elution; solvent selection critical	[45]
SMB	97.8	98.76	80% ethanol/20% water (3.29 L/g)	56.8 mg/h (≈10 × batch)	Lab/preparative scale	complex setup; high cost	[107]
Twin-column recycling chromatography	97.3	99.7	Ethanol	Low injection volume (20 μL); 1.6 mL/mg CBD	Lab/semi-preparative scale	low load; long cycle; high solvent	[108]
Crystallization	≥99 (up to 99.997)	71.98	n-Heptane (recyclable)	n.r.	Lab scale (10 g)	Partial THC removal; strict feed purity (>80% CBD needed)	[110]

Table note: Purity and recovery values depend on feedstock composition, initial CBD content, solvent system, operating scale, and analytical method, and should not be directly compared across studies. “n.r.” indicates that the corresponding data were not reported in the cited literature. Reported scale refers to the experimental scale documented in the cited literature; most studies were conducted at laboratory or pilot scale, and industrial-scale data remain scarce. Target purity and THC limits depend on the intended product category and applicable regulatory framework.

## Data Availability

No new data were created or analyzed in this study. Data sharing is not applicable to this article.
